# Structure-guided design of an LC3B-targeting PNA oligomer modulating autophagy and LC3B-mediated mRNA decay

**DOI:** 10.1039/d6md00523c

**Published:** 2026-08-14

**Authors:** Marco Albani, Enrico Mario Alessandro Fassi, Alessandra Romanelli, Marina Montagnani Marelli, Mariangela Garofalo, Gabriella Roda, Roberta Manuela Moretti, Giovanni Grazioso

**Affiliations:** a Department of Pharmaceutical Sciences, Università degli Studi di Milano Via L. Mangiagalli 25 20133 Milano Italy giovanni.grazioso@unimi.it; b Department of Pharmacological and Biomolecular Sciences, Università degli Studi di Milano Via Balzaretti 9 20133 Milano Italy; c Department of Pharmaceutical and Pharmacological Sciences, Università di Padova Via F. Marzolo 5 35131 Padova Italy

## Abstract

Autophagy plays a central role in cellular degradation and recycling pathways, and the LC3B protein is essential for this process. Dysregulation of LC3B has been implicated in oncogenesis. In this study, we used a computational approach to design a novel peptide nucleic acid (PNA) targeting the RNA-binding domain of LC3B, with the aim of inhibiting its function. The RNA AAUAAA polyadenylation signal was used as a starting point to design new PNAs with high affinity for LC3B. Molecular dynamics simulations and binding free-energy calculations on the RNA-AAUAAA/LC3B complex enabled the identification of promising PNA analogues. The lead candidate, a PNA with the sequence AATAAA, was synthesized, and its biological activity was investigated through biophysical and cellular assays. Cell viability was evaluated in the human prostate cell lines PNT1A and PNT2, as well as in the prostate cancer cell lines PC3 and DU145, while its efficacy in inhibiting autophagy was assessed in PC3 cells. The combined computational, biophysical and cellular results support AATAAA-PNA as an LC3B-binding hit whose cellular effects are consistent with modulation of LC3B-associated autophagy and mRNA-decay pathways. Overall, this study presents an *in silico* strategy for the design and development of LC3B inhibitors based on the RNA AAUAAA motif. The designed PNA represents a promising hit compound for further optimization in cancer therapy.

## Abstract 2

Structure-guided design identified AATAAA-PNA as a low-nanomolar LC3B binder. Cellular effects in PC3 cells were consistent with modulation of autophagy and LC3B-mediated mRNA decay.

## Graphical abstract

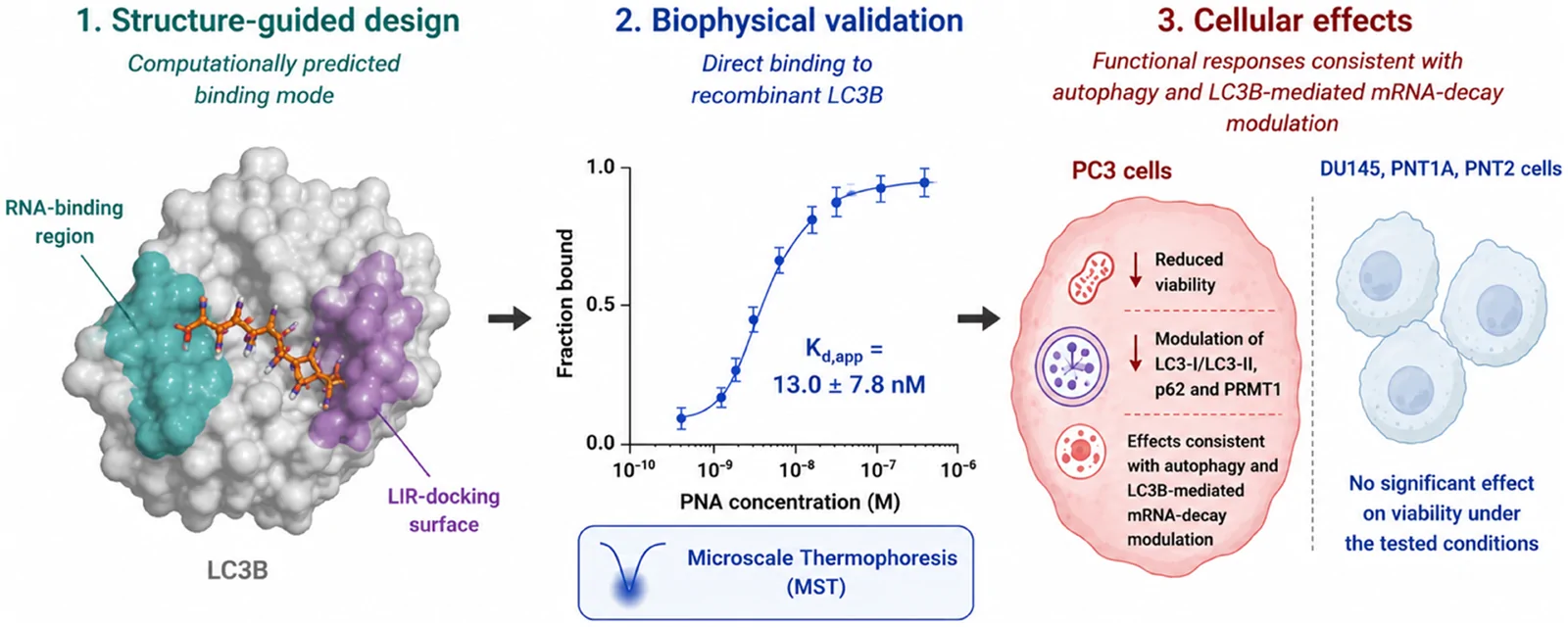

## 1. Introduction

The autophagy process is a meticulously orchestrated mechanism targeting specific proteins and old or damaged organelles using double-membrane vesicles called autophagosomes. After autophagosomes fuse with lysosomes, the engulfed contents are degraded by the acidic environment and lytic enzymes confined within the lysosomes.^1,2^ This recycling process is found in both eukaryotic cells and bacteria, maintaining cell homeostasis and physiological conditions.^2,3^ The autophagy machinery comprises over 50 proteins termed Atgs, with the members of the Atg8 family primarily responsible for cellular trafficking and autophagosome formation. In mammals, Atg8 proteins (mAtg8) consist of two subfamilies: MAP1LC3 (microtubule-associated protein 1 light chain 3), also known as LC3, and GABARAP (GABA-A receptor-associated protein). The LC3 subfamily encompasses LC3A (including two splicing variants, LC3Aα and LC3Aβ), LC3B, LC3B2, and LC3C,^4^ whereas the GABARAP subfamily includes GABARAP, GABARAPL1, and GABARAPL2.

The disruption of the complex autophagy machinery is linked with numerous diseases, such as neurodegenerative disorders,^5^ cardiomyopathies,^6^ infectious diseases,^7,8^ type II diabetes mellitus,^9,10^ hepatic steatosis,^11^ and cancer.^12–14^ Autophagy's functions in cancer are complex and may have conflicting roles in different cancer cells, conditions, and stages. In normal cells, autophagy eliminates misfolded proteins or defective organelles, maintaining cellular health. Consequently, reduced autophagy activity is discerned in the early stages of tumorigenesis. Conversely, autophagy increases during the metastatic stages of cancer, thereby improving tumor cell survival. Furthermore, autophagy is triggered in cancer cells exposed to various stresses, including anticancer medications, leading to chemoresistance.^15,16^

Nonetheless, LC3B is the most widely studied Atg8 protein in humans because it is evidently connected with cancer. Interestingly, the proteins involved in autophagy can interact with LC3B by a specific amino acid sequence referred to as the “LC3 interacting region” (LIR). On the other hand, LC3B interacts with these autophagy proteins through its LIR docking site (LDS), which consists of two hydrophobic pockets, defined as HP1 and HP2. Several peptides were designed to interact strongly with LDS,^17,18^ limiting LC3B action in prostate cancer cells. Recently, LC3B was characterized as an RNA-binding protein (RNA-BP) by its ability to interact with mRNA *via* an arginine-rich motif comprising three arginine residues (Arg68, Arg69, and Arg70), a feature shared with many other RBPs.^19,20^ Specifically, LC3B prefers mRNAs containing the AAUAAA consensus motif, a 3′ polyadenylation signal found in the untranslated region (UTR) of most eukaryotic mRNAs.^21–23^ When LC3B interacts with target mRNAs, it triggers their rapid degradation, a process known as LC3B-mediated mRNA decay (LMD). This mechanism depends on the activation of the CCR4-NOT deadenylase complex, which cleaves the 3′ poly-adenine tail of specific mRNAs, which subsequently undergo degradation. LC3B does not interact with all mRNAs containing the AAUAAA motif,^24^ and additional structural motifs and protein-mediated cues determine substrate selectivity. Among all mRNAs, protein arginine *N*-methyltransferase type 1 mRNA (PRMT1) has recently been identified as a major LMD substrate. This protein acts as a negative regulator of autophagy, and its efficient mRNA degradation *via* LMD facilitates autophagy. In this manner, LMD shapes the transcriptome during early autophagy, eliminating mRNAs and transcripts that could suppress autophagy while creating a positive intracellular environment conducive to cargo degradation.^22^

In this context, LC3B is an autophagy-related target for the development of chemical probes, autophagy-targeting degraders,^25^ and stapled peptides.^26,27^ In parallel, the growing interest in RNA-BPs as pharmacological targets further supports the exploration of the LC3B RNA-binding domain as a noncanonical surface for ligand design.^28^ Together, these observations suggest that LC3B may be approached not only as an autophagy marker but also as a pivotal key protein involved in selective autophagy, RNA recognition, and cancer cell survival.

**Fig. 1:**
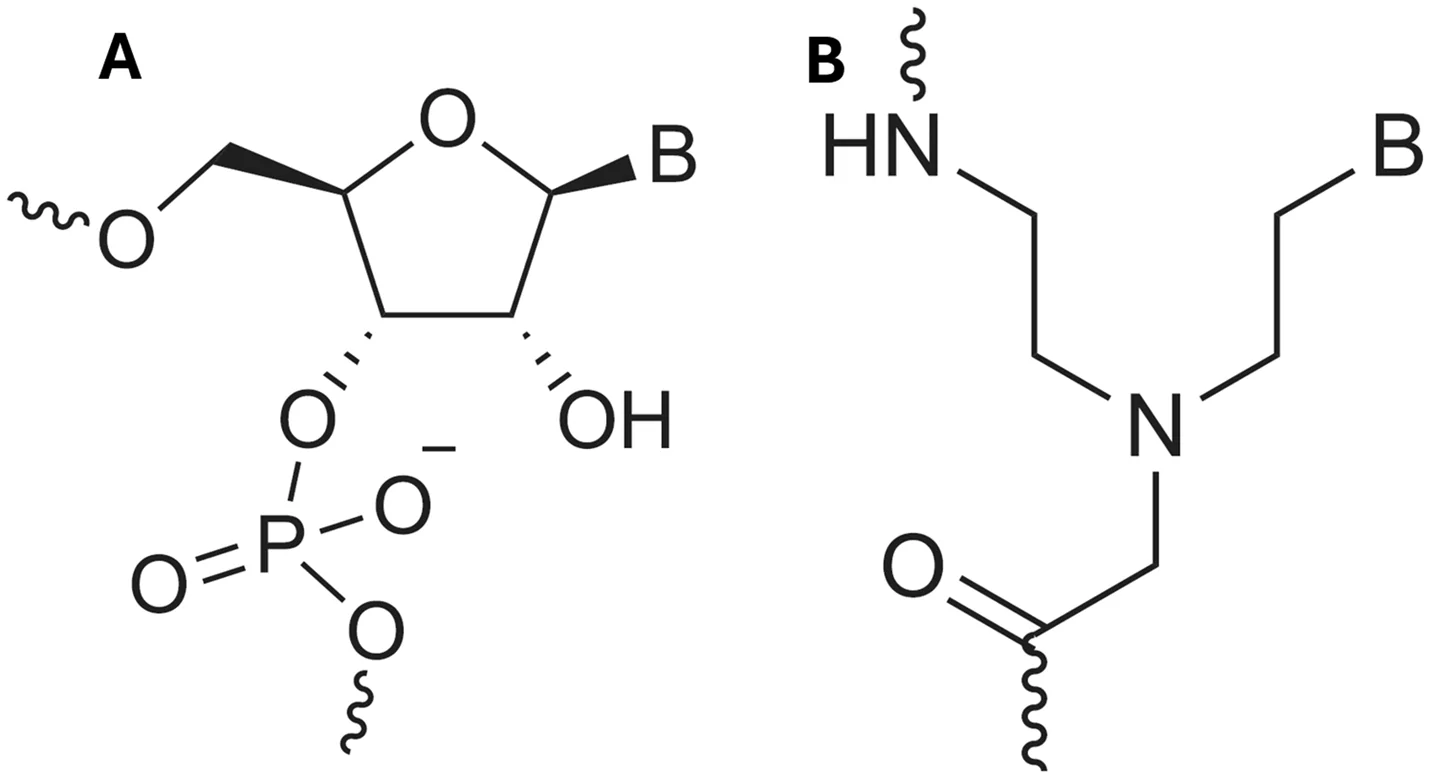
Representation of A) RNA, B) PNA backbones.

In this framework, nucleic acid-based therapeutics and synthetic nucleic acid analogs (xeno nucleic acids, XNAs) offer attractive starting points for developing LC3B-directed ligands. These molecules have expanded the chemical space available for drug discovery, particularly through backbone and nucleobase modifications that improve stability, nuclease resistance, affinity, and target recognition.^29–32^ Among these modalities, PNAs are especially attractive because they combine sequence-programmable recognition with high chemical and enzymatic stability, and chemically modified PNAs have shown increasing potential for RNA-targeted therapeutic applications.^33–35^ However, the clinical translation of PNA-based agents remains limited by cellular uptake and intracellular delivery, prompting the development of peptide-based systems, nanoparticles, liposomes, calixarene-based vectors, and other delivery approaches.^36^ Specifically, PNA is an achiral DNA analog in which the negatively charged phosphodiester backbone is replaced by neutral *N*-(2-aminoethyl)glycine units, thereby conferring resistance to nuclease-mediated degradation.^37^ The nucleobases are bound to these units through a carboxymethylene bridge^38^ (Fig. 1).

Here, we applied a comprehensive computational workflow to develop novel PNA-based mimics of the AAUAAA RNA motif targeting the LC3B RNA-binding domain. The resulting candidates were evaluated in complex with LC3B by molecular dynamics (MD) simulations. The ligand-binding free energy values were predicted using the molecular mechanics-generalized Born surface area (MM-GBSA) approach to select the most promising PNA sequences for subsequent synthesis and testing in biophysical and biological *in vitro* assays on prostate cancer cell lines.

The biological evaluation showed that exposure to the selected PNA reduced PC3 cell viability and modulated LC3, p62, and PRMT1 responses under basal conditions and following autophagy activation by rapamycin, trehalose, or docetaxel. These findings are consistent with modulation of LC3B-associated autophagy and mRNA-decay pathways and support AATAAA-PNA as an initial hit for further characterization and optimization.

## 2. Results and discussion

### 2.1 Computational design of AAUAAA RNA analogues

The identification of LC3B as an RBP opens the way to the design of specific ligands targeting the LC3B arginine-enriched RNA-binding motif to develop a novel class of autophagy modulators. These compounds could selectively interfere with LC3B-mRNA interactions, thereby avoiding LMD in cancer cells and the degradation of its substrates (such as PRMT1 mRNA), while reducing the impact and efficiency of autophagy on cell survival and disrupting LC3B maturation to LC3-II. Consequently, we focused on the rational design of RNA analogs with improved affinity for the LC3B RNA-binding motif, using the polyadenylation signal AAUAAA as a template.

**Fig. 2:**
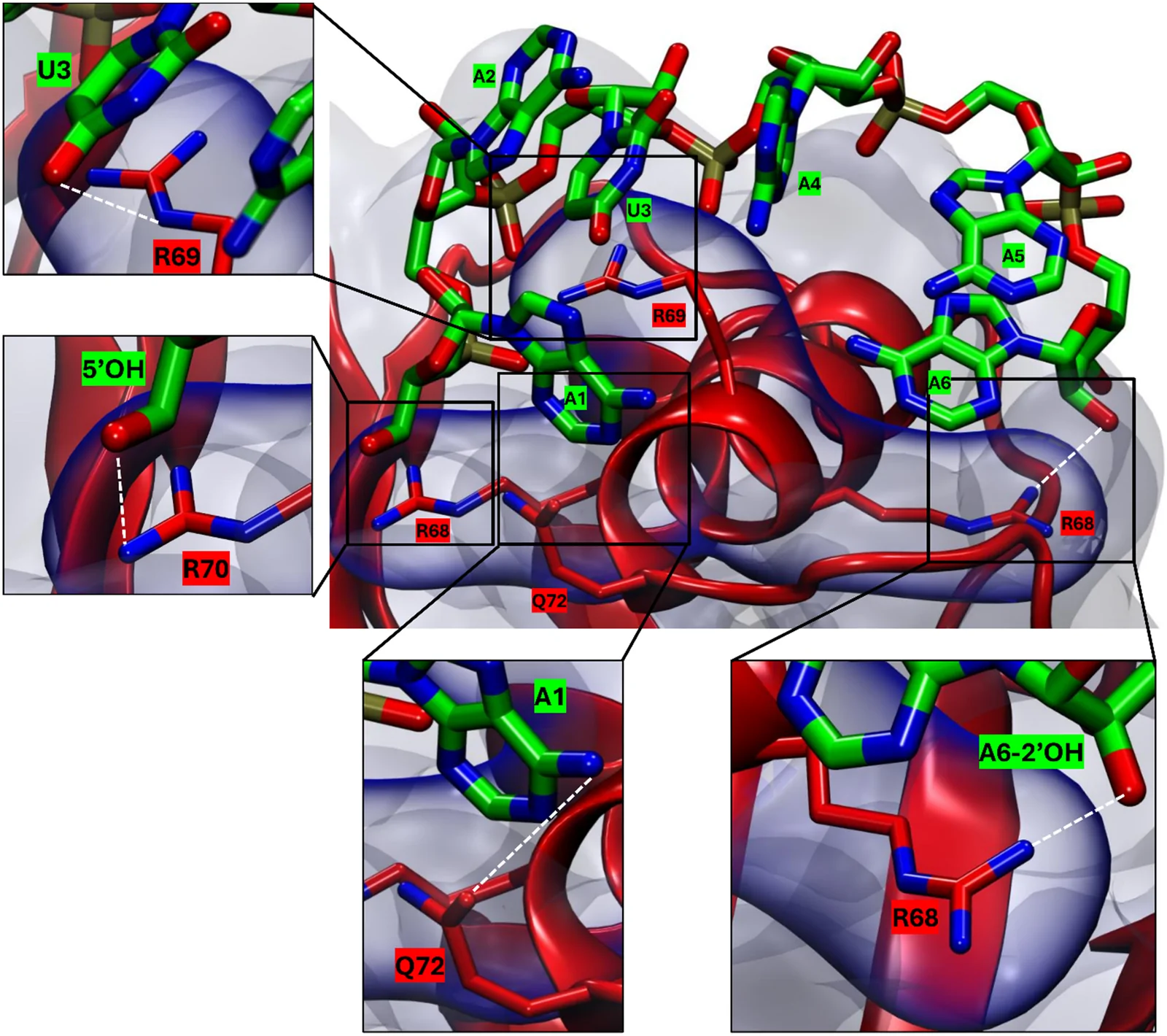
Predicted binding mode of the AAUAAA ssRNA (green licorice representation) in complex with LC3B at the end of the MD simulations. The protein surface is shown in gray, with the RNA-BD shadowed in blue. Key interacting residues (R68, R69, R70, and Q72) are displayed as red sticks. Hydrogen bonds are depicted as white dotted lines. Boxes highlight the interaction between the PNA monomers (green sticks) and the LC3 residue (red sticks).

Specifically, the AAUAAA ssRNA sequence was initially analyzed in complex with LC3B, obtained from docking calculations, by performing energy minimization and MD simulations. When the ssRNA reached conformational stability in complex with LC3B (as ascertained by observing the ligand heavy atoms root mean square deviation (RMSD) plot, Fig. S1, SI), the ssRNA binding free energy value was calculated by applying the MM-GBSA approach, yielding an average value of −151.0 ± 3.3 kJ mol^−1^. This value served as a reference for the subsequent design steps. The visual inspection of the final frame of the LC3B/AAUAAA RNA complex MD trajectory highlighted several key interactions between ssRNA and LC3B (Fig. 2), which could be valuable for the design of novel LC3B binders. In depth, the AAUAAA sequence is primarily stabilized by the arginine-enriched RNA-binding motif constituted of three arginine residues (R68, R69, and R70), which form a strong electrostatic site that positions the RNA phosphate backbone around it, anchoring the structure in place, as highlighted in Fig. 2. Adenine 1 (A1) interacted strongly with LC3B-Q72 by establishing a hydrogen bond, and its amino group binds to the carbonyl oxygen of the LC3B-Q72 side chain, stabilizing the position of the RNA strand over the LC3B RNA-BD. Additionally, the LC3B-R69, another member of the RNA-binding domain, forms a hydrogen-bond-assisted salt bridge with the negatively charged phosphate group of uracil 3 (U3), particularly recruiting one of the non-bridging phosphate oxygens. Furthermore, the U3′ carbonyl oxygen acts as a hydrogen-bond acceptor, interacting with the guanidinium group of LC3B-R69, thereby stabilizing the complex by forming an additional hydrogen-bond-assisted salt bridge (Fig. 2). Together, the latter two interactions provide a strong stabilization of the ssRNA on the surface of LC3B. Moreover, the 5′OH of the AAUAAA RNA sequence forms a hydrogen bond with the guanidinium group of R70. In addition, the 2′OH group of adenine 6 (A6) (Fig. 2) interacts with the guanidinium group of R68, enriching the binding profile of AAUAAA RNA on the LC3B RNA-BP.

### 2.2 Design of novel PNAs

Given the well-known limitations of natural ssRNA as a drug-like molecule, including limited metabolic stability, susceptibility to nuclease degradation, and potential delivery-related drawbacks, we selected PNA as the first XNA scaffold for this study. This choice was guided by three main considerations: first, PNA preserves the nucleobase sequence information of the AAUAAA motif, allowing direct comparison with the native RNA template; second, its neutral backbone removes the negatively charged phosphodiester chain, enabling us to evaluate whether LC3B recognition could be maintained or improved in the absence of phosphate-driven electrostatic interactions; and third, PNA is compatible with rapid solid-phase synthesis and systematic single-base sequence optimization. Therefore, PNA served as a suitable scaffold for exploring whether the AAUAAA recognition motif could be converted into a more stable and chemically tunable LC3B ligand. Moreover, although other XNA backbones may provide comparable advantages in terms of chemical stability and nuclease resistance, PNA was selected as a scaffold for this proof-of-concept study. Therefore, the present work was not intended as a comparative screening of all possible XNA chemistries, but rather as a focused structure-guided investigation of whether the AAUAAA motif could be translated into a neutral and chemically tunable PNA ligand. Future optimization studies will explore whether alternative XNA backbones can further improve LC3B affinity, selectivity, cellular uptake, or biological activity.

Accordingly, we simulated PNA analogs in complex with LC3B alongside the native AAUAAA sequence. The selection process followed a stepwise single-nucleotide substitution strategy focused on the AAUAAA hexamer motif, which functions as the main eukaryotic pre-mRNA polyadenylation signal. To evaluate the impact of single-nucleotide polymorphisms or mutations on binding affinity, we systematically replaced the central uracil (U) with cytosine (C), guanine (G), and adenine (A). The “energetic penalty” of non-canonical bases, which determines the selectivity of the LC3B RNA-binding motif, can be measured through the comparison of binding free energies (Δ*G* values) between these mutants and the wild-type sequence. In parallel, PNA was investigated as a potential scaffolding material for the inhibitors in our study (Table 1). The MM-GBSA method was used to compute the binding affinities of the PNA sequences as Δ*G* values (kJ mol^−1^) for each sequence variant, as previously done for ssRNA. The negative Δ*G* values indicate favorable thermodynamic binding, with more negative values reflecting stronger predicted binding interactions. It should be emphasized that MM-GBSA Δ*G* values are relative estimates, intended for ranking congeneric sequences simulated under an identical protocol, rather than absolute binding free energies directly comparable to experimentally determined values. The approach neglects configurational entropy and explicit solvation contributions and is known to overestimate the magnitude of the computed energies. The values reported in Tables 1–3 should therefore be interpreted as a comparative ranking; the experimental affinity of the selected PNA was determined independently by MST.

**Table 1:** Average Δ*G* values for the RNA and PNA sequences that underwent single-nucleotide substitution. “AATAAA RNA” sequence was not included because thymine is not a canonical RNA nucleobase

Sequence	Avg Δ*G* ± SEM (kJ mol^−1^)
**RNA**	
AAUAAA	−151.0 ± 3.3
AACAAA	−161.1 ± 3.3
AAGAAA	−138.9 ± 3.8
AAAAAA	−203.8 ± 3.8
**PNA**	
AAUAAA	−91.6 ± 1.3
AACAAA	−192.5 ± 1.3
AAGAAA	−122.6 ± 0.8
AAAAAA	−56.1 ± 1.3
AATAAA	−242.7 ± 2.1

**Fig. 3:**
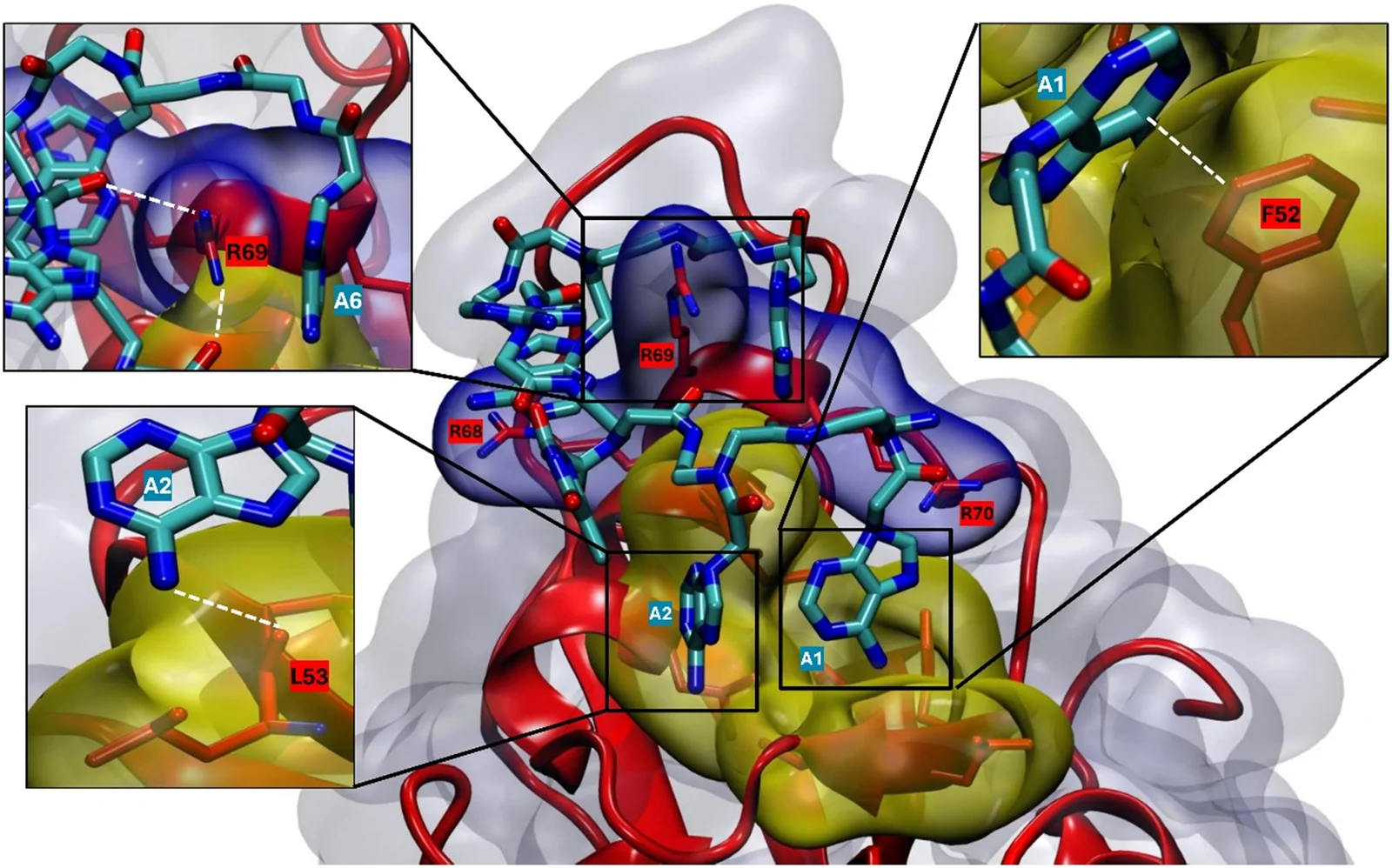
Interaction of the PNA AATAAA sequence (cyan licorice representation) with the LC3B RNA-binding domain (blue transparent surface). The LDS region is highlighted as a yellow transparent surface. LC3B is shown as a gray surface with a red ribbon representation. The interactions are depicted as white dotted lines. Boxes highlight the interaction between the PNA monomers (green sticks) and the LC3 residue (red sticks).

As shown in Table 1, the PNA AATAAA analog exhibited the most favorable binding free energy, −242.7 ± 2.1 kJ mol^−1^ (Fig. 3), approximately 92 kJ mol^−1^ lower than that of the starting ssRNA sequence AAUAAA (−151.0 ± 3.3 kJ mol^−1^). This is particularly interesting because it suggests that 1) each sequence variation produced substantial changes in the predicted binding free energy, specifically regarding the PNA backbone, and 2) only the AATAAA and AACAAA PNA analogs, both containing a pyrimidine in position 3, led to a significant increase in the predicted affinity compared to the RNA AAUAAA sequence.

Based on these results, the PNA AATAAA sequence was further mutated to identify additional sequences that could bind more efficiently. The selection plan adopted in this stage encompassed three distinct categories: (1) modifications to the core motif, entailing single-position substitutions at each nucleotide location of the AATAAA sequence, resulting in 24 variations in which each of the six positions underwent systematic mutation to any of the alternative nucleotides; (2) homopolymeric controls, including sequences entirely comprised of a single nucleotide type (such as AAAAAA, TTTTTT, GGGGGG, CCCCCC, UUUUUU) to establish fundamental binding affinities irrespective of sequence-specific recognition; and (3) 10 randomly designed multiple-point mutated sequences. Overall, 39 different sequences were investigated, as reported in Table 2. This strategy follows established methods for studying nucleic acid ligands by enabling researchers to vary sequence elements for measuring specific nucleotide selection at designated study positions.

**Table 2:** Average Δ*G* values for the PNA sequences, grouped by the most frequent nucleobase in each sequence (thymine, cytosine, guanine, uracil, and adenine), as well as several mixed-base combinations

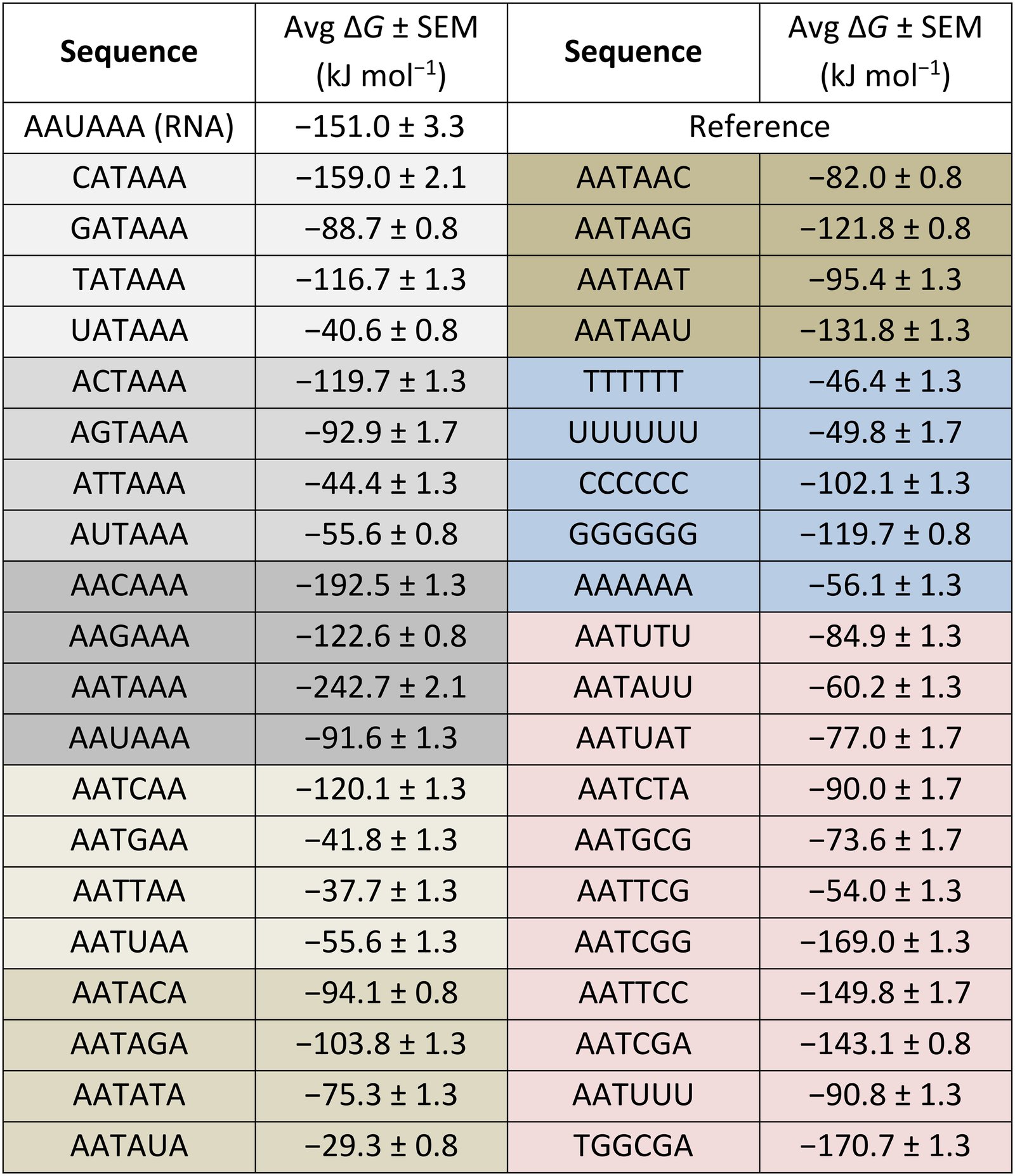

Moreover, this approach allowed us to uncover key elements of the recognition motif through complete saturation analysis of all single-nucleotide variants, without requiring prior identification of functionally relevant positions.^39^ Finally, this method uses homopolymeric controls to distinguish specific-sequence binding effects from those arising from nucleotide-composition preferences.

In the obtained data, the Δ*G* values of the PNA sequences spanned from −29.3 ± 0.8 to −242.7 ± 2.1 kJ mol^−1^, indicating that each nucleobase has a significant impact on the binding stability against the LC3B RNA-BD. The corresponding extremes of this ranking are summarized in Table 3.

**Table 3:** The three most and the three least favorable average Δ*G* values among the simulated PNA sequences, compared with the AAUAAA ssRNA reference

Sequence	Avg Δ*G* ± SEM (kJ mol^−1^)
**Three most favorable PNA sequences**	
AATAAA	−242.7 ± 2.1
AACAAA	−192.5 ± 1.3
TGGCGA	−170.7 ± 1.3
**Three least favorable PNA sequences**	
UATAAA	−40.6 ± 0.8
AATTAA	−37.7 ± 1.3
AATAUA	−29.3 ± 0.8
**Reference**	
AAUAAA (RNA)	−151.0 ± 3.3

Specifically, even minor sequence variations, such as uracil substitutions to thymine or adenine to guanine, significantly altered the predicted binding affinity. Furthermore, all other sequence optimizations applied to the starting AATAAA sequence did not improve the ligand binding free energy, suggesting that this motif is critical for binding to LC3B RNA-BD and for performing LMD. The visual inspection of the trajectory frame attained at the end of MD simulations revealed that the particularly favorable binding mode adopted by PNA with sequence AATAAA accounted for its lowest predicted Δ*G* value (Fig. 3).

More specifically, the neutral charge of the PNA backbone was responsible for the markedly different binding mode, compared with the natural AAUAAA RNA sequence. Indeed, our simulations suggest that LC3B-F52 engages in a π–π stacking interaction with the adenine ring of A1, positioning the nucleotide within a deep LDS pocket implicated in modulating autophagy (Fig. 3). The carbonyl group of the PNA backbone between A5 and A6, as highlighted in Fig. 3 (top left box), interacts with the guanidinium group of LC3B-R69, thereby stabilizing the ligand. Finally, the amine group of PNA-A2 interacted with the carboxyl group of the LC3B-L53 backbone, creating a hydrogen bond. Then, PyContact^40^ was used to identify and characterize the interactions between the ssRNA and PNA sequences and LC3B.

**Fig. 4:**
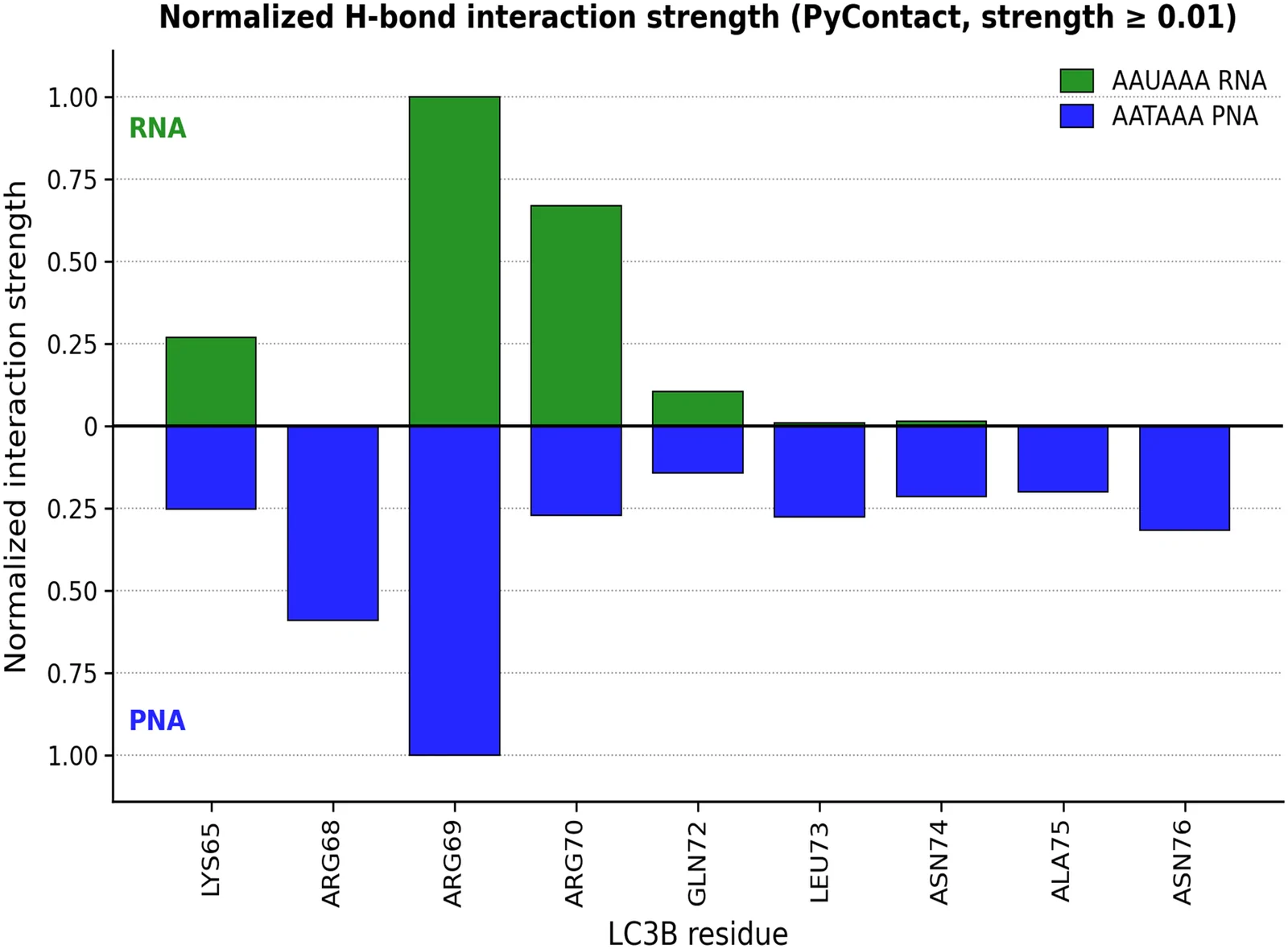
Normalized hydrogen-bond interaction strength between LC3B and the ssRNA AAUAAA (green, plotted upwards) or the PNA AATAAA (blue, plotted downwards), computed with PyContact.^40^ For each LC3B residue, the bar height reports the normalized contacts count of the hydrogen bonds formed with the ligand. Within each series, values are normalized to the strongest interaction of that series; the comparison therefore refers to the shape of the two interaction profiles rather than to absolute contact numbers. Residues whose normalized strength was below 0.01 in both series are not shown.

This analysis aimed to clarify which nucleotides and LC3B key residues can provide the most effective interactions. The results highlighted that the strongest interactions were the hydrogen bonds between the ssRNA AAUAAA and R69 and R70. For the PNA AATAAA, the profile was similar, involving R69, R68, and N76, as highlighted in Fig. 4. A complementary per-residue contact-occupancy analysis, computed as the fraction of MD frames in which each LC3B residue lies within 5.0 Å of the ligand, confirmed that both molecules engage the arginine-rich RNA-BD, whereas the PNA additionally contacts residues in and around the LDS, including K30, I35, D48, K49, F52, L53, V54, P55, H57, and V58 (Fig. S2, SI).

**Fig. 5:**
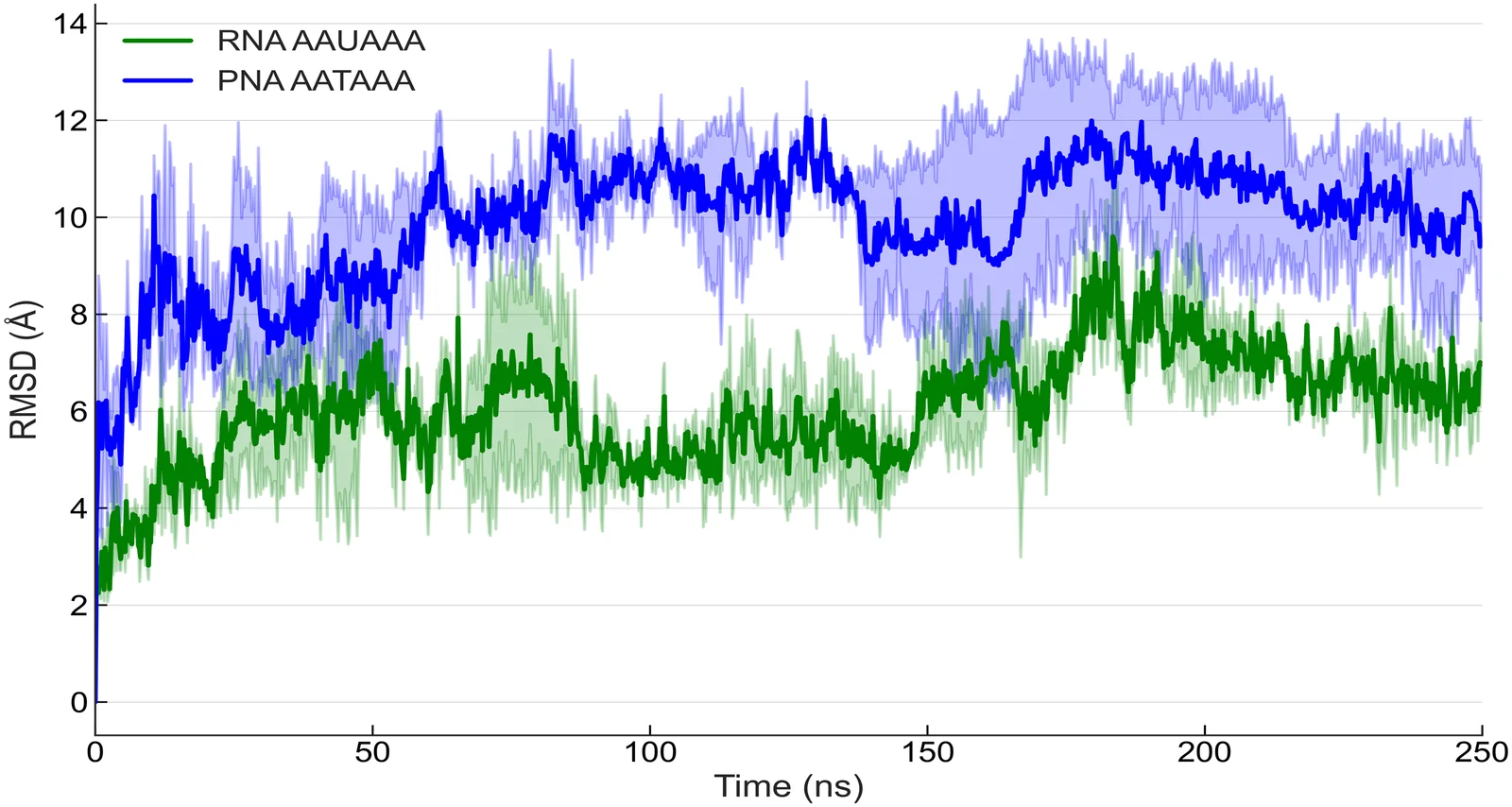
RMSD *vs.* MD simulation time. In green, the RNA AAUAAA sequence, while in blue, the PNA AATAAA sequence. The standard deviation is depicted as a shade for each point in time.

The RMSD plot analysis of the ssRNA AAUAAA and PNA AATAAA sequences (Fig. 5) highlights a similar overall trend. Specifically, the AAUAAA ssRNA exhibits a more stable profile, showing an average RMSD value of approximately 7 Å relative to the docking pose. This stabilization occurs when the negatively charged phosphate backbone of the RNA interacts with the LC3B RNA-binding domain. In contrast, the neutrally charged PNA stabilizes at RMSD values close to 10 Å relative to the starting pose, as shown in Fig. 5. The different binding mode established by the PNA AATAAA achieved a lower binding free energy. However, a key difference was the deeper penetration of AATAAA into the hydrophobic pocket, allowing for additional hydrophobic contacts with LC3B-F52 and -L53. Notably, this different placement of the ligand does not perturb the protein: in both complexes, LC3B retains a stable backbone RMSD, a constant radius of gyration, and an unchanged content of ordered secondary structure (Fig. S3, SI), and the distributions of these descriptors computed over the equilibrated portion of the trajectories (125–250 ns) are largely superimposable (Fig. S4, SI). The per-residue Cα RMSF analysis further indicates that the PNA systematically dampens the fluctuations of the RNA-BD, particularly in the N74–A78 flanking loop, while preserving the global flexibility profile of LC3B (Fig. S5, SI).

Although the present work focuses on LC3B, future profiling against LC3A, LC3C, GABARAP, GABARAPL1, and GABARAPL2 will be required to define the selectivity profile of PNA AATAAA within the human Atg8 family.

In conclusion, computational studies suggested that the PNA AATAAA analog can emulate the key interaction patterns of the RNA AAUAAA while establishing a distinct interaction network on the LC3B surface. Although the native RNA AAUAAA sequence is strongly stabilized by phosphate-mediated electrostatic interactions with the arginine residues of the RBD, the PNA AATAAA analog appears to compensate for a partial loss of these interactions through a different set of nucleobase-mediated contacts. In addition, the conformational adaptability of the PNA scaffold enables the nucleobases to adopt conformations that maximize binding interactions through spatial complementarity in both RNA-BD and LDS. Consistently, throughout the simulations, the AATAAA PNA displays a higher internal RMSF and a more compact shape, in terms of both end-to-end distance and radius of gyration, than the natural AAUAAA ssRNA (Fig. S6, SI). Accordingly, the superposition of the LC3B-bound AAUAAA and AATAAA poses shows that the two ligands occupy a common region of the LC3B surface, with the PNA scaffold partially displaced toward the LDS (Fig. S7, SI). Nevertheless, CD spectroscopy and thermal-stability analyses would be required for a complete experimental characterization of the effects of PNA binding on LC3B structural stability.

Overall, the neutral and chemically tunable PNA scaffold represents a suitable backbone for developing a more metabolically stable ligand. To conclude, throughout our simulations, we have identified a novel PNA analog endowed with improved predicted binding affinity, enhanced stability on the LC3B surface, and high metabolic stability.

### 2.3 Biophysical assays

**Fig. 6:**
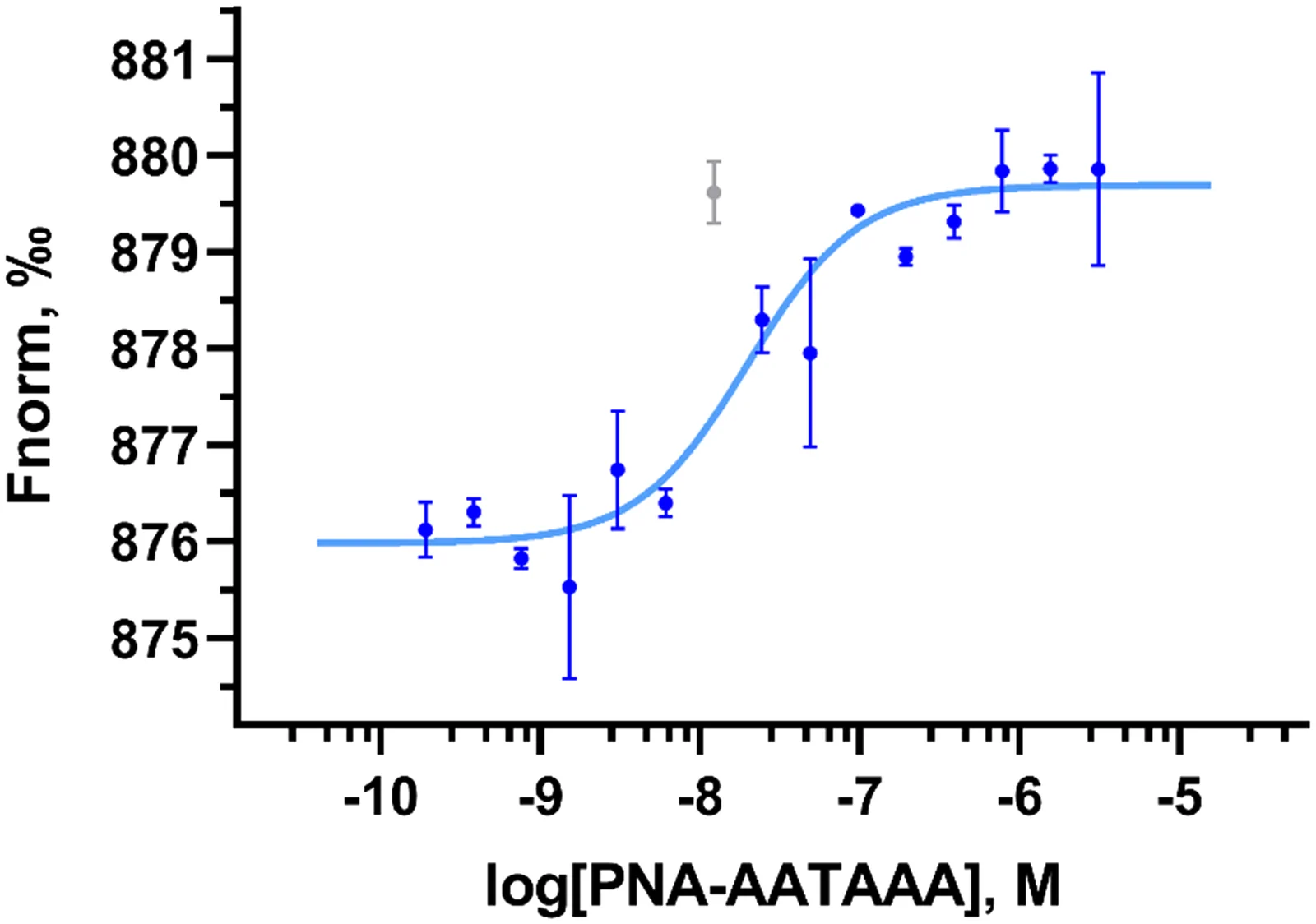
MST binding curve acquired using human recombinant His-tagged LC3B protein incubated with different concentrations of the PNA AATAAA using the Monolith NT.115 instrument. The first concentration point (6.2 μM) was excluded because it fell completely outside the measurable range, while the ninth point (12.2 nM), identified as a clear outlier, is shown in grey. Both points were omitted from the Kd calculation.

To assess whether PNA AATAAA can modulate the autophagy machinery through direct binding to LC3B, we performed Microscale Thermophoresis (MST) experiments using human recombinant His-tagged LC3B. This solution-based technique monitors changes in the thermophoretic behavior of a fluorescently labeled molecule upon application of a temperature gradient. Binding of a non-fluorescent ligand alters key physicochemical properties, such as charge, size, or hydration shell, resulting in measurable shifts in thermophoretic mobility. Because MST operates entirely in the liquid phase, it avoids artifacts associated with surface immobilization.^41^ MST measurements revealed a tight binding of PNA AATAAA to LC3B protein, displaying an apparent dissociation constant (*K*_d_) value of 13.0 ± 7.8 nM (Fig. 6). Experimental conditions are described in the Experimental section while the full MST analysis report of PNA AATAAA is available in the SI, Fig. S8.

### 2.4 Biological activity of PNA AATAAA on prostate cancer cells

To evaluate the biological impact of PNA AATAAA, hereafter PNA, we first assessed its effect on cell viability in PC3 cells. PNA AATAAA was administered by direct addition of the unmodified oligomer to the complete culture medium. No transfection reagent, cell-penetrating peptide, nanoparticle, or other delivery system was used; therefore, cells were treated under gymnotic conditions. Cells were treated with PNA for 24 and 48 hours at increasing doses from 1 nM to 1 μM, and an MTT assay was performed. The PNA was soluble at the stock and working concentrations used in the cellular assays, with no visible precipitation during preparation or treatment.

**Fig. 7:**
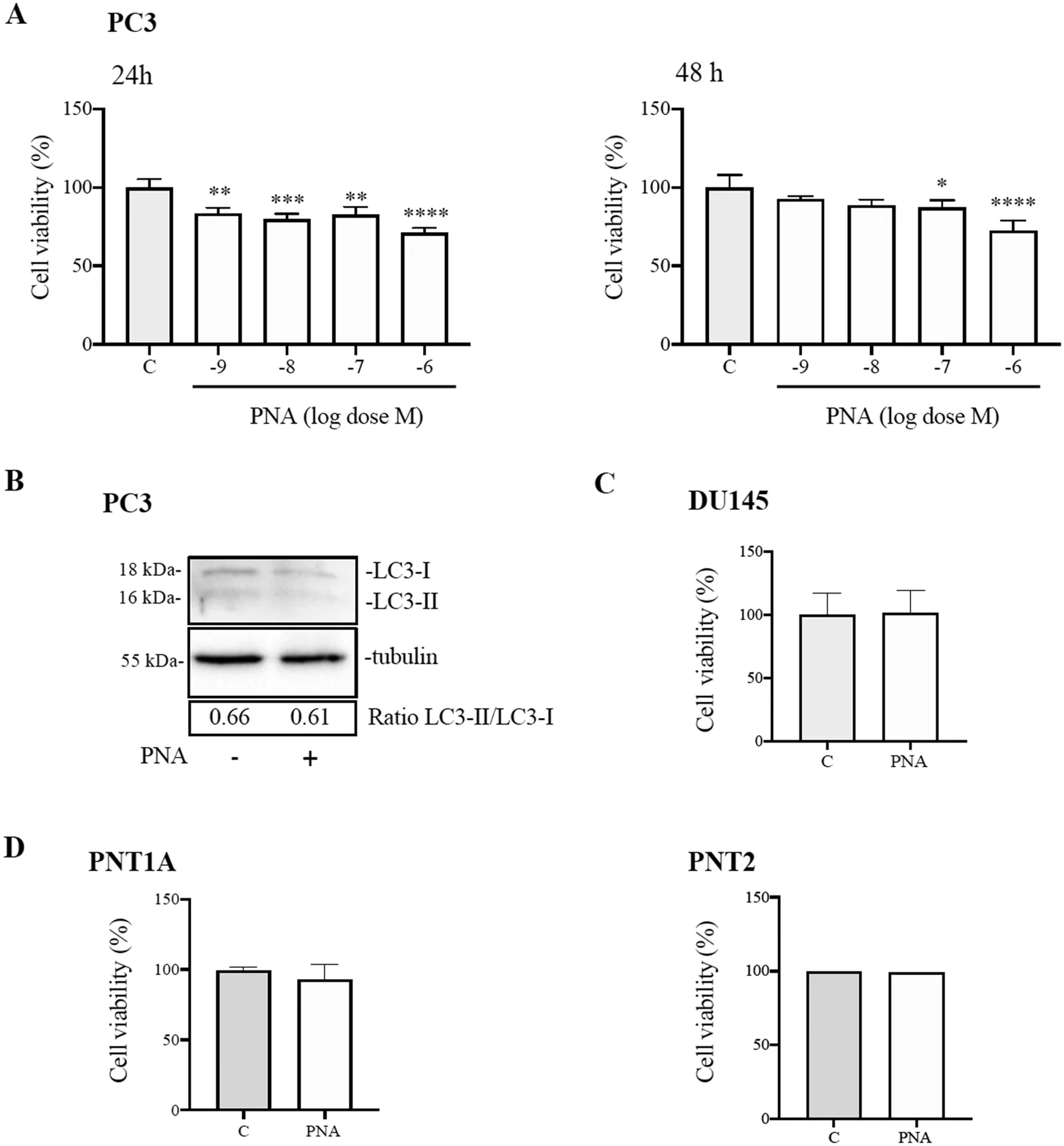
Effect of PNA AATAAA on CRPC cells and normal prostate cells. (A) Cell viability was determined by MTT assay after 24 and 48 h of treatment with PNA AATAAA (PNA) on PC3 cells. Four independent biological samples were analyzed for each condition (*n* = 4). Statistical analysis was performed using one-way analysis of variance (ANOVA) followed by Dunnett's *post hoc* test (* *p* < 0.05 *vs.* C; ** *p* < 0.01 *vs.* C; *** *p* < 0.001 *vs.* C; **** *p* < 0.0001 *vs.* C). (B) Western blot of LC3 in PC3 cells treated with and without PNA (1 μM, 48 h). Tubulin expression was evaluated as a protein control. (C) Cell viability was determined by MTT assay after treatment with PNA (1 μM, 48 h) on DU145 cells. Four independent biological samples were analyzed for each condition (*n* = 4). Statistical analysis was performed using one-way analysis of variance (ANOVA) followed by an unpaired *t*-test. (D) Cell viability assays were performed after treatment with PNA on PNT1A (1 μM, 48 h) and PNT2 (1 μM, 96 h) cells. Four (PNT1A) or three (PNT2) independent biological samples were analyzed for each condition (*n* = 4; *n* = 3). Statistical analysis was performed using one-way analysis of variance (ANOVA) followed by an unpaired *t*-test.

The results shown in Fig. 7A indicate that exposure to PNA reduces PC3 cell viability in a concentration-dependent manner. After 24 h of treatment, PNA induced a significant effect at concentrations ranging from 1 nM to 1 μM compared with control cells containing vehicle (DMSO); in contrast, after 48 h, significant effects were observed only at the highest concentrations tested, 0.1 and 1 μM. The lower effect observed at the lowest concentrations after 48 h may reflect time-dependent adaptive cellular responses or differences in the kinetics of the cellular response; the present experiments do not allow discrimination among these possibilities. Accordingly, subsequent experiments were performed using PNA at 1 μM for 48 h. These findings prompted us to investigate whether the reduction in PC3 cell viability could be associated with modulation of basal autophagy. LC3B expression was therefore evaluated by Western blot analysis. No significant change in the LC3-II/LC3-I ratio was observed between control and PNA-treated cells. However, both LC3-I and LC3-II levels were reduced following PNA treatment, suggesting that PNA may affect basal autophagy and LC3B processing (Fig. 7B).

To further investigate the possible contribution of the autophagic machinery to the cellular response, we evaluated PNA activity in DU145 cells, which harbour an ATG5 alteration and display impaired autophagy.^18,42^ PNA treatment (1 μM, 48 h) did not significantly affect DU145 cell viability (Fig. 7C). This result is consistent with a contribution of an intact autophagic machinery to the response observed in PC3 cells, although alternative mechanisms cannot be excluded.

Furthermore, we evaluated the effect of PNA on cell viability in two prostatic epithelial cell lines (PNT1A and PNT2 cells). Treatment with PNA for 48 h at a 1 μM dose did not exert any effect on PNT1A cells, as well as the treatment (1 μM, 96 h) on PNT2 cells (Fig. 7D). PNA also did not significantly affect the viability of the non-tumoral prostate epithelial cell lines PNT1A and PNT2 under the tested conditions (Fig. 7D). Taken together, this cell-dependent response argues against a uniform, cell-line-independent cytotoxic effect, although it does not by itself demonstrate intracellular LC3B target specificity or exclude additional cellular targets.

### 2.5 Effect of PNA on autophagy activation in PC3 cells

The conversion of LC3B from the unconjugated form (LC3-I) to the PE-conjugated form (LC3-II) represents a fundamental step for autophagy activation and the correct formation of autophagosomes.^43^ In addition, autophagy activation promotes the association of LC3B with mRNA targets and induces rapid degradation of these transcripts. This process, known as LMD (LC3B-mediated mRNA decay), targets the mRNA of PRMT1, a negative regulator of autophagy. Rapid degradation of PRMT1 mRNA by LMD contributes to the progression of autophagy.^21^ Because LC3B-mediated binding and degradation of specific mRNAs occur only following activation of the autophagic response, we examined this process using three distinct autophagy activators: rapamycin, trehalose, and docetaxel.

Rapamycin is an mTOR inhibitor that activates autophagy in PC3 cells, decreasing cell viability after treatment at doses from 10 to 100 nM for 48 h (Fig. S9A, SI). The activity of rapamycin determines the activation of autophagy with the presence of an active autophagic flux.^43^

Trehalose is a disaccharide known to activate autophagy in an mTOR-independent manner by inducing an increase in LC3 and p62 expression and leading to the accumulation of autophagosomes that are only partially eliminated by the autophagic flux.^43^ The treatment for 48 h in doses from 50 to 100 mM does not affect PC3 cell viability (Fig. S9B, SI).

Docetaxel is a drug that alters microtubule dynamics. The treatment for 48 h in doses from 10 to 100 nM induces a significant reduction of cell viability (Fig. S9C, SI). In response to this stress, cells activate an autophagic response, as evidenced by the LC3 expression in PC3 cells treated with 25 nM docetaxel (Fig. S9D, SI).

We first analyzed PNA's ability to inhibit rapamycin-induced autophagy through the evaluation of LC3-I and LC3-II expression. PC3 cells were treated with rapamycin (100 nM, 48 h) with or without PNA (1 μM). The result shows that PNA does not alter the LC3-II/LC3-I ratio, although both LC3-I and LC3-II levels were reduced compared with the control. Rapamycin increased the LC3-II/LC3-I ratio, as expected given its role as an autophagy activator.

**Fig. 8:**
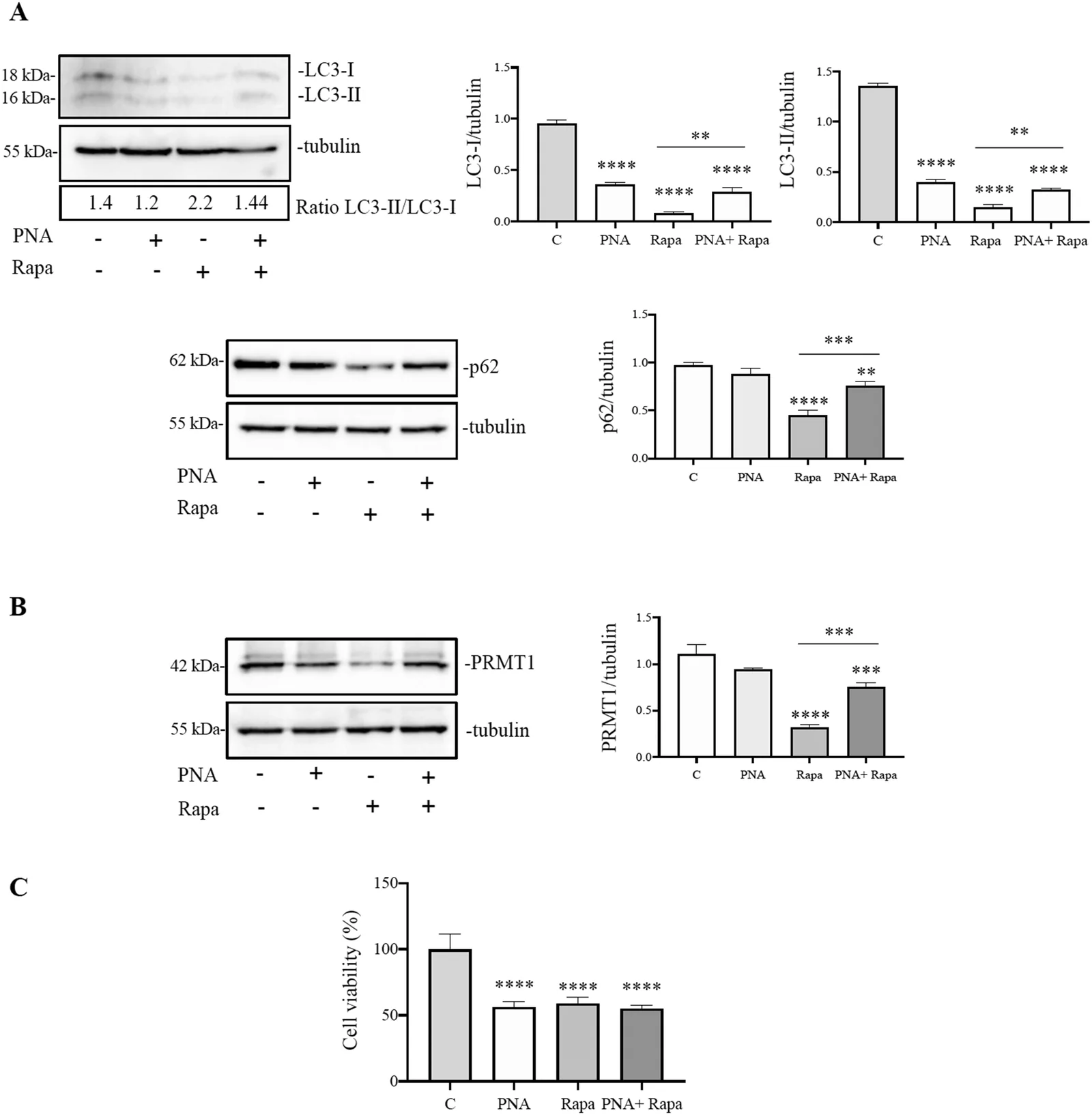
Effect of PNA AATAAA on rapamycin-induced autophagy in PC3 cells. (A) Western blot of both LC3 and SQSTM1 (p62) was performed in cells treated with PNA AATAAA (PNA) (1 μM, 48 h) and rapamycin (100 nM, 48 h) (Rapa). Tubulin expression was evaluated as a protein control. Three independent biological samples for each condition were analyzed (*n* = 3), bar graph represents the mean optical density ± SD. Statistical analysis of LC3-I and LC3-II expression was performed using One-way ANOVA followed by Tukey's *post hoc* test (**** *p* < 0.0001 *vs.* C; ** *p* < 0.01). Statistical analysis of p62 expression was performed using One-way ANOVA followed by Tukey's *post hoc* test (** *p* < 0.01 *vs.* C; **** *p* < 0.0001 *vs.* C; *** *p* < 0.001). (B) Western blot of PRMT1 was analyzed in cells treated with PNA (1 μM, 48 h) and Rapa (100 nM, 48 h). Tubulin expression was evaluated as a protein control. Three independent biological samples for each condition were analyzed (*n* = 3), bar graph represents the mean optical density ± SD. Statistical analysis was performed using One-way ANOVA followed by Tukey's *post hoc* test (* *p* < 0.05 *vs.* C; *** *p* < 0.001 *vs.* C; *** *p* < 0.0001 *vs.* C; *** *p* < 0.001). (C) Cell viability was determined by MTT assay after treatment with PNA (1 μM, 48 h) and Rapa (100 nM, 48 h). Four independent biological samples were analyzed for each condition (*n* = 4). Statistical analysis was performed using One-way ANOVA followed by Tukey's *post hoc* test (**** *p* < 0.0001 *vs.* C).

PNA combined with rapamycin inhibits rapamycin-induced autophagy activation, restoring LC3-I and LC3-II levels to control values, confirming PNA counteraction on the autophagy activated by rapamycin (Fig. 8A).

We then analyzed the expression of SQSTM1/p62 (p62), a selective autophagy receptor that recognizes cellular cargo and targets it for autophagosomal degradation. Its expression levels reflect the rate at which material within autophagosomes is delivered to lysosomes for degradation. p62, indeed, allows for investigating autophagic flux.

WB analysis of p62 shows that PNA does not alter p62 expression; rapamycin induces a reduction of p62 expression, confirming its ability to activate significant autophagic flux; PNA appears to counteract rapamycin-induced p62 decrease (Fig. 8A).

LC3B has been shown to bind PRMT1 mRNA following autophagy activation, leading to its degradation.^21,22^ We therefore assessed PRMT1 expression after rapamycin treatment (100 nM) with or without PNA (1 μM). WB of PRMT1 shows that rapamycin causes a marked reduction in the protein; although PNA alone did not significantly affect PRMT1 expression, it counteracted the rapamycin-induced reduction in PRMT1 expression (Fig. 8B). These results suggest that PNA interferes with LC3B-mediated mRNA decay, likely by occupying or perturbing the LC3B RNA-binding region, thereby limiting rapamycin-induced downregulation of PRMT1. We therefore tested the effect of combined rapamycin and PNA treatment on PC3 cell viability. We treated for 48 h with PNA at 1 μM with or without rapamycin (100 nM). The assay confirms that PNA exerts an antitumor effect; rapamycin, as expected, inhibits cell viability; and PNA counteracts the inhibitory activity of rapamycin (Fig. 8C).

**Fig. 9:**
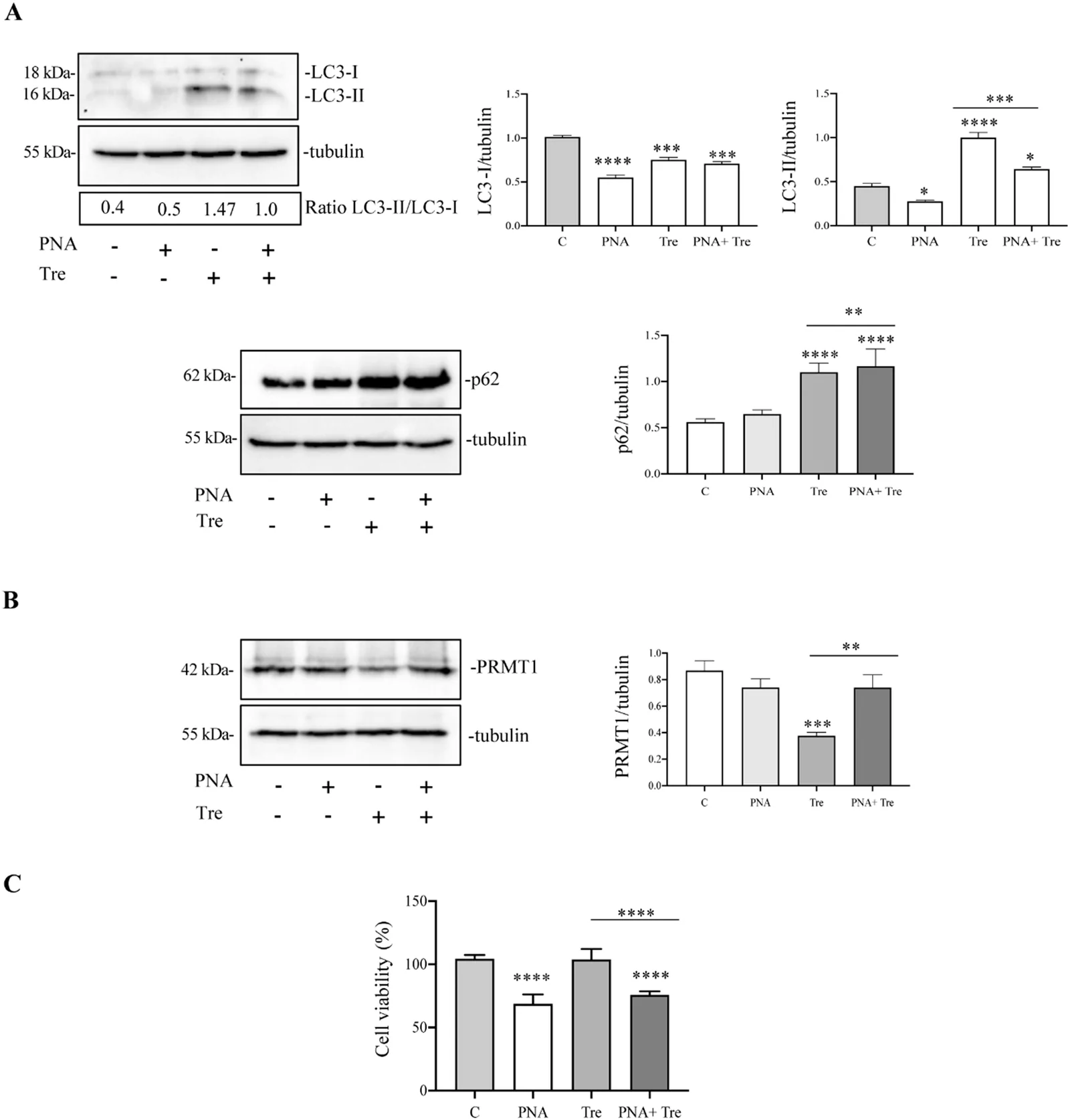
Effect of PNA AATAAA on trehalose-induced autophagy in PC3 cells. (A) Western blot of both LC3 and SQSTM1 (p62) was performed in cells treated with PNA AATAAA (PNA) (1 μM, 48 h) and trehalose (100 mM, 48 h) (Tre). Tubulin expression was evaluated as a protein control. Three independent biological samples for each condition were analyzed (*n* = 3), bar graph represents the mean optical density ± SD. Statistical analysis of LC3-I and LC3-II expression was analyzed using One-way ANOVA followed by Tukey's *post hoc* test (* *p* < 0.05 *vs.* C; *** *p* < 0.001 *vs.* C; **** *p* < 0.0001 *vs.* C; *** *p* < 0.001). Statistical analysis of p62 expression was analyzed using One-way ANOVA followed by Tukey's *post hoc* test (**** *p* < 0.0001 *vs.* C; ** *p* < 0.01). (B) Western blot of PRMT1 was analyzed in cells treated with PNA (1 μM, 48 h) and trehalose (100 mM, 48 h). Tubulin expression was evaluated as a protein control. Three independent biological samples for each condition were analyzed (*n* = 3), bar graph represents the mean optical density ± SD. Statistical analysis was performed using One-way ANOVA followed by Tukey's *post hoc* test (*** *p* < 0.001 *vs.* C; ** *p* < 0.01). (C) Cell viability was determined by MTT assay after treatment with PNA (1 μM, 48 h) and trehalose (100 mM, 48 h). Four independent biological samples were analyzed for each condition (*n* = 4). Statistical analysis was performed using One-way ANOVA followed by Tukey's *post hoc* test (**** *p* < 0.0001 *vs.* C; **** *p* < 0.0001).

We then analyzed the PNA activity on trehalose-activated autophagy. WB of LC3 shows that PNA (1 μM, 48 h) counteracts the increase in the LC3-II/LC3-I ratio induced by trehalose (100 mM), demonstrating that the inhibitor can counteract trehalose-induced autophagy (Fig. 9A).

The analysis of p62 shows that PNA does not alter protein expression; in contrast, trehalose induces a significant increase. This result is known in fact, trehalose increases p62 transcription, and the synthesized protein is only partially degraded at the lysosomal level. This impairment in autophagic flux leads to increased levels of p62.^43^ In this context, PNA is unable to counteract the increase in p62. Fig. 9B shows that treatment with trehalose (100 mM, 48 h) significantly reduced PRMT1 expression. This result highlights that trehalose also triggers LMD as rapamycin. PNA does not affect PRMT1 expression but reverts the trehalose-induced reduction of PRMT1 expression. Analysis of cell viability confirms PNA's ability to inhibit tumor growth, and this action is maintained even in the presence of trehalose, which does not alter cell viability (Fig. 9C).

**Fig. 10:**
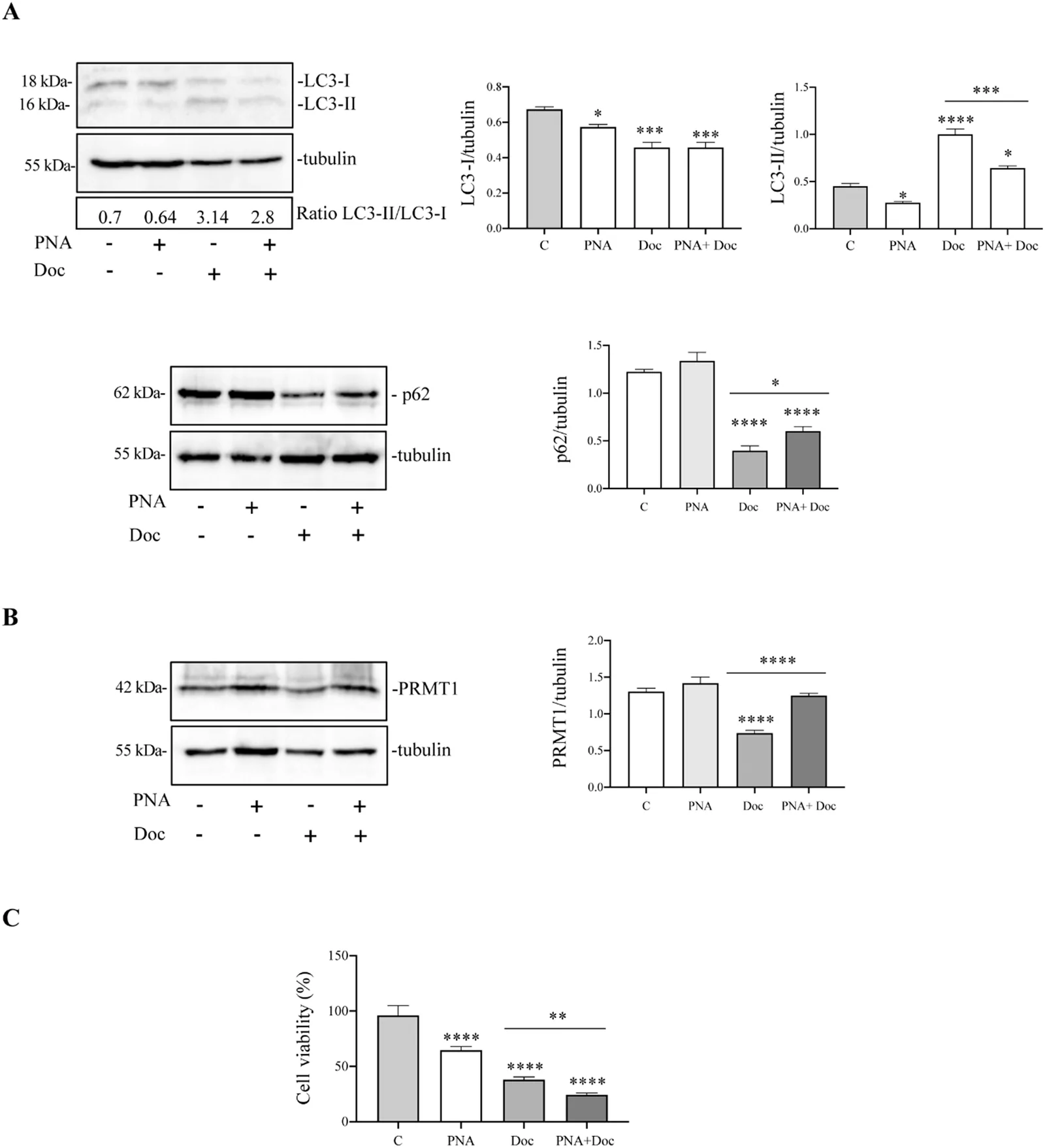
Effect of PNA AATAAA on docetaxel-induced autophagy in PC3 cells. (A) Western blots of both LC3 and p62 were performed in cells treated with PNA AATAAA (PNA) (1 μM, 48 h) and docetaxel (25 nM, 48 h) (Doc). Tubulin expression was evaluated as a protein control. Three independent biological samples for each condition were analyzed (*n* = 3); the bar graph represents the mean optical density ± SD. Statistical analysis of LC3-I and LC3-II was conducted using One-way ANOVA followed by Tukey's *post hoc* test (* *p* < 0.05 *vs.* C; *** *p* < 0.001 *vs.* C; **** *p* < 0.0001 *vs.* C; *** *p* < 0.001). Statistical analysis of p62 expression was performed using One-way ANOVA followed by Tukey's *post hoc* test (** *p* < 0.01 *vs.* C; **** *p* < 0.0001 *vs.* C; *** *p* < 0.001). (B) Western blot of PRMT1 in cells treated with PNA (1 μM, 48 h) and Doc (25 nM, 48 h). Tubulin expression was evaluated as a protein control. Three independent biological samples for each condition were analyzed (*n* = 3); the bar graph represents the mean optical density ± SD. Statistical analysis was performed using One-way ANOVA followed by Tukey's *post hoc* test (**** *p* < 0.0001 *vs.* C; * *p* < 0.05; **** *p* < 0.0001). (C) Cell viability was determined by MTT assay after treatment with PNA (1 μM, 48 h) and Doc (25 nM, 48 h). Four independent biological samples were analyzed for each condition (*n* = 4). Statistical analysis was performed using One-way ANOVA followed by Tukey's *post hoc* test (**** *p* < 0.0001 *vs.* C; ** *p* < 0.01).

Finally, we analyzed the role of PNA in combination with docetaxel. Many studies have been conducted to understand whether treatment with docetaxel could induce autophagy in prostate cancer cells and whether this activation enhances or counteracts the efficacy of chemotherapy. The analysis of LC3 expression confirms that docetaxel (25 nM, 48 h) increased the LC3-II/LC3-I ratio, and combined treatment with PNA (1 μM) brought this ratio to baseline (Fig. 10A). Assessment of p62 expression indicated that docetaxel activates autophagy and enhances autophagic flux, as reflected by decreased p62 levels. While PNA alone did not significantly alter p62 expression, co-treatment with PNA and docetaxel impaired the docetaxel-induced autophagic response, resulting in p62 accumulation. We then evaluated the effect of the combined treatment of PNA and docetaxel on PRMT1 expression. Fig. 10B shows that docetaxel (25 nM, 48 h) causes a reduction of PRMT1 expression, and PNA co-treatment partially reverses this effect, supporting an effect consistent with inhibition of the LMD response activated by docetaxel. We then analyzed the effect of PNA on cell viability post-docetaxel treatment. As shown in Fig. 10C, both PNA and docetaxel significantly decreased cell viability, and their combination further potentiated this effect. These findings are consistent with the hypothesis that PNA counteracts the protective autophagic response induced by docetaxel, thereby enhancing the docetaxel-associated reduction in PC3 cell viability.

The results obtained are consistent with numerous studies that have explored the role of autophagy in modulating the cellular response to chemotherapy.^44^

In this context, the use of autophagy inhibitors in combination with chemotherapy represents an attractive strategy to enhance the anticancer activity of conventional therapeutic agents. Accordingly, an increasing number of autophagy inhibitors have been identified and investigated.

Autophagy can be pharmacologically inhibited at different stages of the autophagic process, including early initiation steps and late events such as autophagosome–lysosome fusion and cargo degradation. The mechanisms of the major autophagy inhibitors proposed for anticancer therapy have been extensively studied in preclinical *in vitro* and *in vivo* models, as well as in clinical trials, often in combination with pharmacological treatments.^45^

However, many of these inhibitors remain nonspecific, affecting multiple molecular pathways, whereas others act primarily by blocking late autophagic flux, as observed for chloroquine and hydroxychloroquine.^46–47^ To better contextualize the LC3B-directed strategy proposed in this study, Table 4 summarizes representative autophagy inhibitor classes, their main mechanisms of action, their principal limitations, and the distinctive features of the PNA AATAAA scaffold.

**Table 4:** Representative classes of autophagy inhibitors investigated in cancer therapy and comparison with the LC3B-directed AATAAA-PNA described in this study^17,18,25–27,45–47^

Inhibitor class	Representative examples	Main autophagy stage/target	Main limitation
Lysosomotropic autophagy inhibitors	Chloroquine (CQ), hydroxychloroquine (HCQ)	Late flux/lysosome	Low specificity; toxicity
PI3K/VPS34 pathway inhibitors	Wortmannin, 3-methyladenine, SAR405	Autophagy initiation	Broad PI3K pathway effects
ULK1/ULK2 inhibitors	SBI-0206965, MRT68921, DCC-3116	ULK kinase complex	Kinase selectivity; compensation
LC3/GABARAP-directed peptides	RavZ, FYCO1-derived and stapled LIR peptides	LIR-docking site	Peptide delivery and stability
LC3/GABARAP small-molecule ligands	GW5074-related ligands; LC3/GABARAP fragments	LC3/GABARAP binding pockets	Early-stage chemical probes
Small nucleic acid binders	AATAAA-PNA	LC3B RNA-BD/LDS	Delivery, stability, and selectivity

Taken together, the cellular findings are consistent with modulation of early autophagic events and of the LC3B-associated LMD response. The computational study predicts that AATAAA-PNA may engage an extended LC3B surface involving the RNA-binding region and residues adjacent to the LDS, whereas MST confirms direct binding to recombinant LC3B. However, the contribution of these individual binding regions and direct intracellular target engagement were not fully experimentally resolved. Our data suggest that PNA could play a dual role, inhibiting both basal pro-survival autophagy and rapamycin-, trehalose-, and docetaxel-induced autophagy, and this second function is exerted by interfering with LC3B's ability to bind PRMT1 mRNA and promote its degradation. Overall, these findings suggest that PNA may act as a novel autophagy inhibitor capable of potentiating therapeutic cytotoxicity in settings where cytoprotective autophagy contributes to treatment resistance.

## 3. Conclusions

Our computational approach aimed at designing new PNA sequences endowed with high affinity to LC3B. This study initially focused on the ssRNA AAUAAA sequence, a polyadenylation signal found in several LC3B-binding mRNAs. A computational workflow combining MD simulations and MM-GBSA calculations was then applied to predict the binding affinity of novel candidates generated through backbone and sequence optimization.

As depicted in Tables 1 and 2 and Fig. 2 and 3, our methodology enabled the design of a PNA ligand with enhanced affinity to LC3B compared to the natural RNA. The hydrogen bonds and electrostatic interactions between the novel PNA sequence and LC3B's positively charged hydrophobic surface, defined by residues R68, R69, R70, F52, and L53, result in a low binding free energy, indicating improved binding efficiency. Furthermore, the backbone modification conferred flexibility and neutral charge while improving metabolic stability, allowing the sequence to target the RNA-BD and LDS simultaneously. The robustness of this approach is supported by binding experiments together with biological and biophysical analyses, confirming that PNA AATAAA exerts anticancer activity by inhibiting cytoprotective autophagy in tumor cells. Accordingly, the observed biological effects, which are consistent with the modulation of LC3B-associated RNA decay and autophagy, provide a promising basis for the design of engineered PNA sequences incorporating unnatural nucleobases to further improve the binding affinity and cellular activity of the AATAAA motif. Under these circumstances, the development of an artificial intelligence model capable of predicting the most stable and biologically active sequences from combinations of natural and unnatural nucleobases could drastically hasten the identification and optimization of next-generation PNA candidates. Overall, this work provides a structure-guided framework for developing LC3B-directed PNA ligands and supports PNA AATAAA as a promising hit compound for future optimization as an autophagy-modulating agent.

## 4. Experimental section

### 4.1 Computational design of AAUAAA RNA analogues

The LC3B computational model used in this study was constructed using the 3D coordinates of chain A of the LC3B complex, retrieved from the Protein Data Bank (PDB accession code 1V49 (ref. 48)). The original LC3B sequence, which consists of 125 amino acids, undergoes the removal of amino acids 121 to 125 from the C-terminus. Therefore, the computational model used in this project represents the pro-LC3B state of LC3B (amino acids 1 to 120), hereinafter referred to as LC3B for simplicity. The model was optimized using the Protein Preparation tool in Maestro (release 2024-1, Schrödinger, LLC, New York, NY, USA), which included the following: residue protonation state assignment at pH 7.4, residue verification, clash resolution, and optimization of the internal H-bond network. The reference ssRNA, with sequence AAUAAA, was constructed by means of the Build tool of Maestro (Schrödinger Inc., USA, version 2024-1), while the LC3B/AAUAAA RNA complex was generated using the molecular docking software HDOCK.^49^ The protein–ssRNA complex was solvated in a cubic water box with a minimum distance of 10 Å between the complex and the box boundaries. After the addition of an initial Cl^−^ counterion, the system was energy-minimized and subsequently neutralized with the appropriate counterions. To relieve steric clashes, a multi-step minimization protocol was applied, consisting of steepest-descent minimization followed by conjugate-gradient minimization. The system was then gradually heated from 50 to 300 K over 20 ps under constant-volume conditions and equilibrated for 20 ps at 1 atm under constant-pressure conditions to stabilize the system density. Finally, two independent MD simulation replicas were carried out.

These simulations were completed under constant temperature and pressure conditions, 300 K and 1 atm, respectively, using the Langevin thermostat^50^ and Monte Carlo barostat.^51^ The force fields used during these simulations were ff19SB^52^ for LC3B, OL3 (ref. 53) for the RNA, and TIP3P^54^ for the water molecules. GAFF2 was used for the unnatural backbone PNA.^55^ Moreover, the MM-GBSA method^56^ was employed to estimate the ligand-binding free energy for each replica in each system. MM-GBSA energies were used exclusively for the relative ranking of the simulated sequences and are not directly comparable to experimentally measured binding free energies. The analysis was conducted on representative frames extracted from the stabilized portion of each MD trajectory. Specifically, the frame selection was performed by visual inspection of the MD simulations themselves and by analyzing the ligand RMSD *vs.* time plot. After MD simulations, the frame representative of the most populated cluster, defined as the cluster centroid, was selected through the AMBER24/cpptraj cluster analysis tool. The cluster centroid was extracted only from the AAUAAA RNA trajectories to obtain a starting reference structure, which was used for backbone and sequence optimization by simulating PNA analogs.^57^ Regarding the RNA/PNA/LC3B contact analysis, PyContact was run with a distance cutoff of 5.0 Å for general contacts, a donor–acceptor distance cutoff of 2.5 Å for hydrogen bonds, and an angular cutoff of 120° to ensure geometrically meaningful interactions.

### 4.2 Synthesis of the PNA AATAAA

The PNA oligomer (AATAAA) was assembled on a Rink Amide resin or TG XV RAM resin, using standard protocols for Fmoc chemistry.^58^ The oligomer was acetylated at the N-terminus. The product was analyzed by RP-HPLC on a C18 Sepachrom Vydamas column (150 × 4.6 mm) and purified on a Phenomenex Jupiter 10 μm Proteo 90 Å 100 × 21.2 mm column using a gradient of CH3CN (0.1% TFA) in water (0.1% TFA) from 5 to 50% in 20 minutes. The pure compound was characterized by LC-MS (Fig. S10, SI). Purified products underwent repeated lyophilization cycles to remove residual counterions. Calculated mass: 1701.64 u, found: 1702.06, 851.83, 568.42, 426.58.

### 4.3 MST experiments

The interaction between PNA AATAAA and LC3B was examined using a Monolith NT.115 instrument (NanoTemper Technologies, Munich, Germany). Human recombinant His-tagged LC3B (14555-H07E; Sino Biological, Beijing, China) was labeled with the RED-tris-NTA 2nd Generation His-Tag kit (MO-L018; NanoTemper Technologies) for 30 min at room temperature and analyzed using the “binding affinity” mode implemented in the software MO.Control (version 1.6; NanoTemper Technologies). A constant concentration of labeled LC3B (35 nM) was incubated with sixteen 1 : 1 serial dilutions of PNA AATAAA (from 6.2 μM to 0.191 nM). Both components were prepared in PBS containing 0.05% Tween™ 20 and 2.5% DMSO for molecular biology (D8418; Sigma-Aldrich, Saint Louis, USA). After 15 min of incubation at room temperature, samples were loaded into standard capillaries (MO-K022; NanoTemper Technologies) and measured with 80% excitation power and 40% MST power (medium) at 25 °C. Two independent experiments were performed to compute the *K*_d_ curve. Signal evaluation at 5 s yielded a response amplitude of 3.7 (above the 2.5 binding threshold) and a signal-to-noise ratio of 9.7 (above the 5.0 threshold). The binding curve was fitted using the software MO.Affinity Analysis (version 2.3; NanoTemper Technologies) and the figure was generated with the software GraphPad Prism (version 8.0.2; GraphPad, Boston, MA, USA).

### 4.4 Cell cultures

PC3 and DU145 human CRPC cell lines were purchased from the American Type Culture Collection (ATCC, USA). PNT2 (European Collection of Authenticated Cell Cultures, ECACC, UK) human prostate cell line was purchased from Sigma-Aldrich. PNT1A human cell line was established by immortalization of prostate epithelial cells with SV40.^59^

PC3 and DU145 cells were cultured at 37 °C and 5% CO_2_ in RPMI 1640 (EuroClone, Milano, Italy) supplemented with 7.5% (PC3) and 5% (DU145) FBS (Gibco Laboratories, USA). PNT1A and PNT2 were cultured at 37 °C and 5% CO_2_ in RPMI 1640 supplemented with 10% FBS.

### 4.5 Cell viability assays

AATAAA-PNA was dissolved in DMSO at a stock concentration of 10 mM and administered by direct addition to the complete culture medium without any transfection reagents or delivery systems. Cells were therefore treated under gymnotic conditions. The final DMSO concentration was (0.01% v/v) and was kept constant in the corresponding vehicle-treated control conditions.

#### MTT assay

PC3, DU145, and PNT1A cells were plated at a density of 3 × 104 cells per well (PC3 and DU145) and 2 × 104 cells per well (PNT1A) into 24-well plates. After 48 h, PC3 cells were treated with PNA at 0.001 μM, 0.01 μM, 0.1 μM and 1 μM doses for 24 and 48 h.

In combination studies, PC3 cells were treated with PNA (1 μM) and rapamycin (100 nM) (Sigma-Aldrich, St. Louis, MO, USA, R0395) or trehalose (100 mM) (Sigma-Aldrich, T0167) and/or docetaxel (25 nM) for 48 h.

PNT1A and DU145 cells were treated with PNA at a 1 μM dose. At the end of treatments, the medium was replaced with 3-(4,5-dimethylthiazole-2-yl)-2,5-diphenyltetrazolium bromide (MTT) solution (Sigma-Aldrich) (0.5 mg ml^−1^) in RPMI without phenol red and FBS. After 30–45 min at 37 °C, the formazan precipitate was dissolved in isopropanol. The absorbance (550 nm) was measured by an EnSpire Multimode Plate reader (Perkin Elmer, Milano, Italy). Untreated cells were set at 100% (control), and the viability of treated cells was expressed as a percentage of the control. The experiments were independently performed three times, and each treatment was performed in quadruplicate (*n* = 4).

#### MTS assay

PNT2 cells were seeded at a concentration of 1 × 104 cells per well into 96-well plates and cultured overnight under standard conditions. Cells were then treated with PNA at a concentration of 1 μM. Untreated cells were used as a control. 96 h post-treatment, cell viability was determined using CellTiter 96 AQueous One Solution Cell Proliferation Assay (MTS) according to the manufacturer's instructions (Promega, Madison, WI, USA). The absorbance was measured with a 96-well plate spectrophotometer (Victor Nivo TM, PerkinElmer, Milan, Italy) at 490 nm. The experiments were independently performed three times, and each treatment was performed in triplicate (*n* = 3).

### 4.6 Western blot (WB)

PC3 cells were plated at 2 × 105 cells per dish in 6 cm dishes, treated for 48 h, and adherent and floating cells were harvested and lysed in RIPA buffer. Protein extracts (15–25 μg) were resuspended in reducing Sample buffer (Bio-Rad Laboratories, Segrate, Milano, Italy), heated at 95 °C for 5 min, and separated by electrophoretic SDS-PAGE WB. Proteins were transferred onto nitrocellulose or PVDF membranes. After blocking, membranes were incubated with anti-LC3 (1 : 1000, Sigma-Aldrich, L8918), anti-SQSTM1/p62 (1 : 2000, Thermo Fisher Scientific, PA5-20839), anti-PRMT1 (1 : 1000, Upstate-Merck, 07-404) primary antibodies overnight at 4 °C. Peroxidase-conjugated secondary anti-rabbit or anti-mouse antibodies were used for 1 h at room temperature, and the membranes were processed using the enhanced chemiluminescence kit Cyanagen Ultra (Cyanagen, Bologna, Italy). In each WB experiment, β-tubulin expression (1 : 1000, Sigma-Aldrich, T4026) was evaluated as a protein control. Relative optical density of the bands was assessed by ImageJ software. Uncropped WB images are available in SI.

### 4.7 Statistical analysis

Statistical analysis of the results was performed by one-way analysis of variance (ANOVA) followed by an unpaired *t*-test, Dunnett's test or Tukey's multiple-comparison post-test. Prism software was used for the analyses (Prism 8 for Mac OS version 8.2.1, GraphPad Software, San Diego, CA, USA).

## Author contributions

Marco Albani: investigation, methodology, validation, writing – original draft, writing – review & editing. Enrico Mario Alessandro Fassi: conceptualization, investigation, methodology, writing – original draft. Alessandra Romanelli: investigation, methodology, writing – original draft. Marina Montagnani Marelli: investigation, writing – original draft. Mariangela Garofalo: investigation, writing – original draft. Gabriella Roda: funding acquisition, methodology, project administration. Roberta Manuela Moretti: conceptualization, funding acquisition, methodology, supervision, writing – review & editing. Giovanni Grazioso: conceptualization, funding acquisition, methodology, project administration, supervision, writing – review & editing.

## Conflicts of interest

The authors declare that they have no known competing financial interests or personal relationships that could have appeared to influence the work reported in this paper.

## Supplementary Material

MD-017-D6MD00523C-s001

## Data Availability

The data supporting the findings of this study are included in the article and in the accompanying supplementary information (SI). Additional data not included in these files are available from the corresponding authors upon request. Supplementary information: Fig. S1–S10: the ligand heavy atoms RMSD *vs.* time plots (Fig. S1); the per-residue LC3B–ligand contact occupancy (Fig. S2); the structural stability and flexibility analyses of LC3B in complex with the AAUAAA ssRNA and the AATAAA PNA (Fig. S3–S5); the conformational descriptors of the two ligands and the structural overlap of their bound poses (Fig. S6 and S7); the full MST binding report of PNA AATAAA (Fig. S8); the characterization of the autophagy activators used in the cellular assays (Fig. S9); and the HPLC profile and the MS spectrum of the tested PNA (Fig. S10). SI is available from the website. See DOI: https://doi.org/10.1039/d6md00523c.
