# Supplementary material for: Structure-guided design of an LC3B-targeting PNA oligomer modulating autophagy and LC3B-mediated mRNA decay
Source: RSC Med Chem. 2026 Aug 14. Online ahead of print. doi: 10.1039/d6md00523c (PMC13496138; doi:10.1039/d6md00523c)
Supplement: Supplementary file 1 [file MD-OLF-D6MD00523C-s001.pdf]

Supplementary Information (SI) for RSC Medicinal Chemistry.
This journal is © The Royal Society of Chemistry 2026

Supporting Information

Structure-Guided Design of an LC3B-Targeting PNA Oligomer Modulating Autophagy and LC3B-Mediated mRNA Decay

Marco Albani[1], Enrico Mario Alessandro Fassi[1], Alessandra Romanelli[1], Marina Montagnani Marelli[2], Mariangela Garofalo[3], Gabriella Roda[1], Roberta Manuela Moretti[2,*], and Giovanni Grazioso[1,*]

[1] Department of Pharmaceutical Sciences, Università degli Studi di Milano, Via L. Mangiagalli 25, 20133 Milano, Italy;

[2] Department of Pharmacological and Biomolecular Sciences, Università degli Studi di Milano, Via Balzaretti 9, 20133 Milano, Italy;

[3] Department of Pharmaceutical and Pharmacological Sciences, Università di Padova, Via F. Marzolo 5, 35131 Padova, Italy;

* These authors contributed equally.

Correspondence: giovanni.grazioso@unimi.it, roberta.moretti@unimi.it

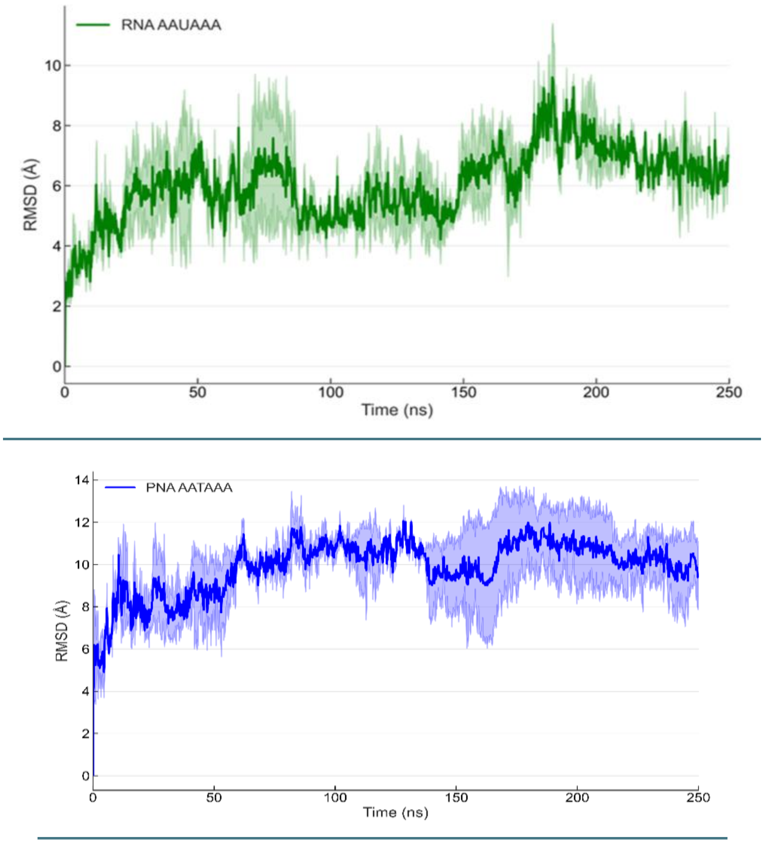

Figure S1: Average ligand heavy atoms RMSD vs. Time plots of RNA AAUAAA (green) and PNA AATAAA (blue) over 250 ns of MD simulation time.

For Figures S2–S7, values are reported as means over independent MD replicas when applicable, and dispersion is the standard deviation between replicas. Colour coding follows the main computational figures: green, AAUAAA ssRNA; blue, AATAAA PNA.

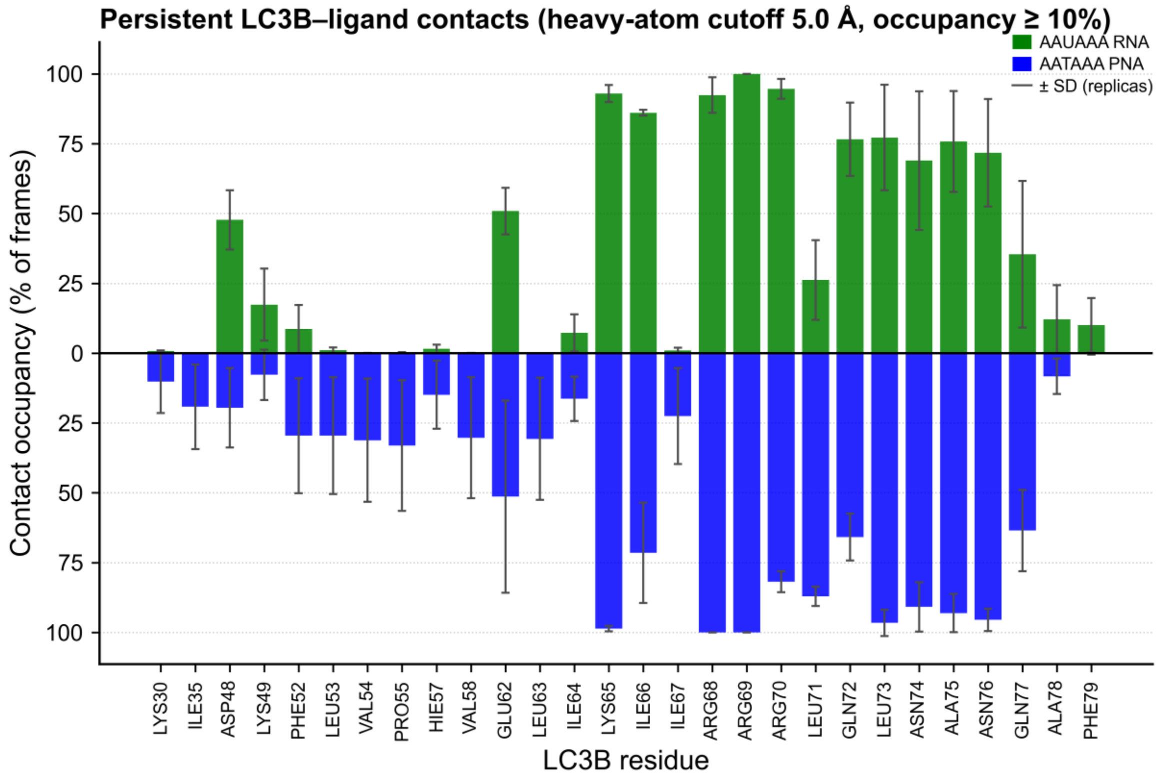

Figure S2. Per-residue LC3B-ligand contact occupancy during the MD simulations. For each residue, the plotted value is the fraction of frames in which any heavy atom lies within 5.0 Å of any ligand heavy atom; AAUAAA ssRNA contacts are plotted upwards in green and AATAAA PNA contacts downwards in blue. Bars report the mean over replicas and error bars the standard deviation between replicas. Both ligands engage the arginine-rich RNA-binding motif, whereas the PNA additionally contacts residues in and around the LIR-docking site, including K30, I35, D48, K49, F52, L53, V54, P55, H57, and V58.

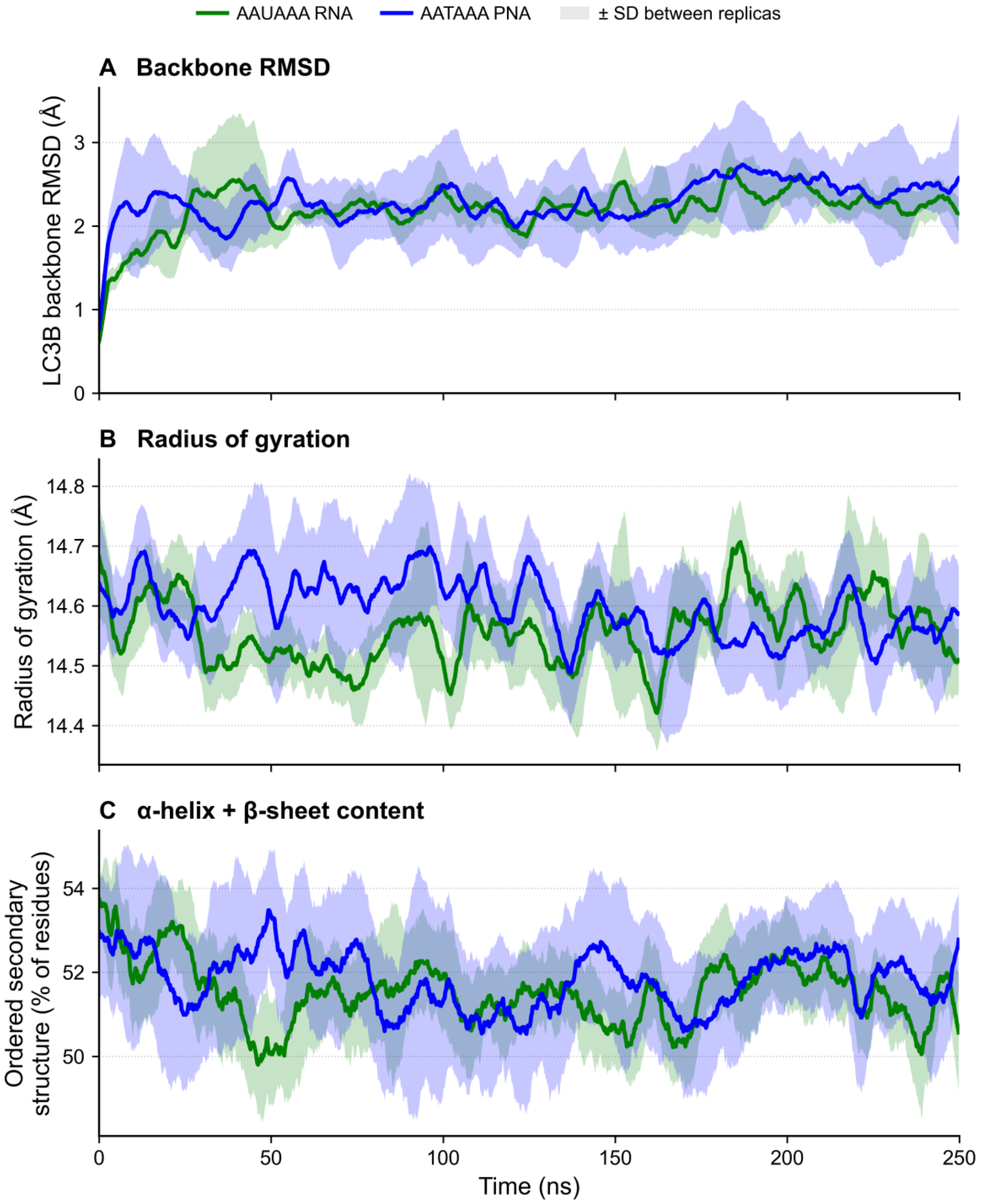

Figure S3. Structural stability of the LC3B fold in complex with AAUAAA RNA (green) and AATAAA PNA (blue) over 250 ns of MD simulation. (A) Backbone RMSD from the energy-minimized starting structure, (B) protein radius of gyration, and (C) fraction of residues assigned to ordered secondary structure by DSSP. Solid lines report the mean over independent replicas and shaded areas the standard deviation between replicas, with a 5 ns running mean applied for clarity. Both complexes remain folded and compact, indicating that PNA binding does not destabilize the LC3B.

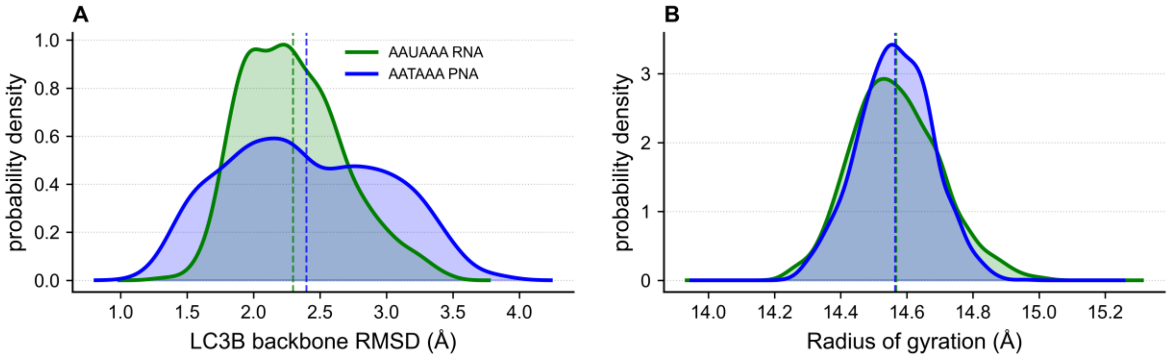

Figure S4. Distribution of global LC3B structural descriptors over the equilibrated portion of the trajectories (125–250 ns). (A) Backbone RMSD and (B) radius of gyration are shown as kernel density estimates with all replicas pooled; dashed vertical lines mark the means. The largely overlapping unimodal distributions indicate that LC3B samples the same folded conformational basin with either ligand.

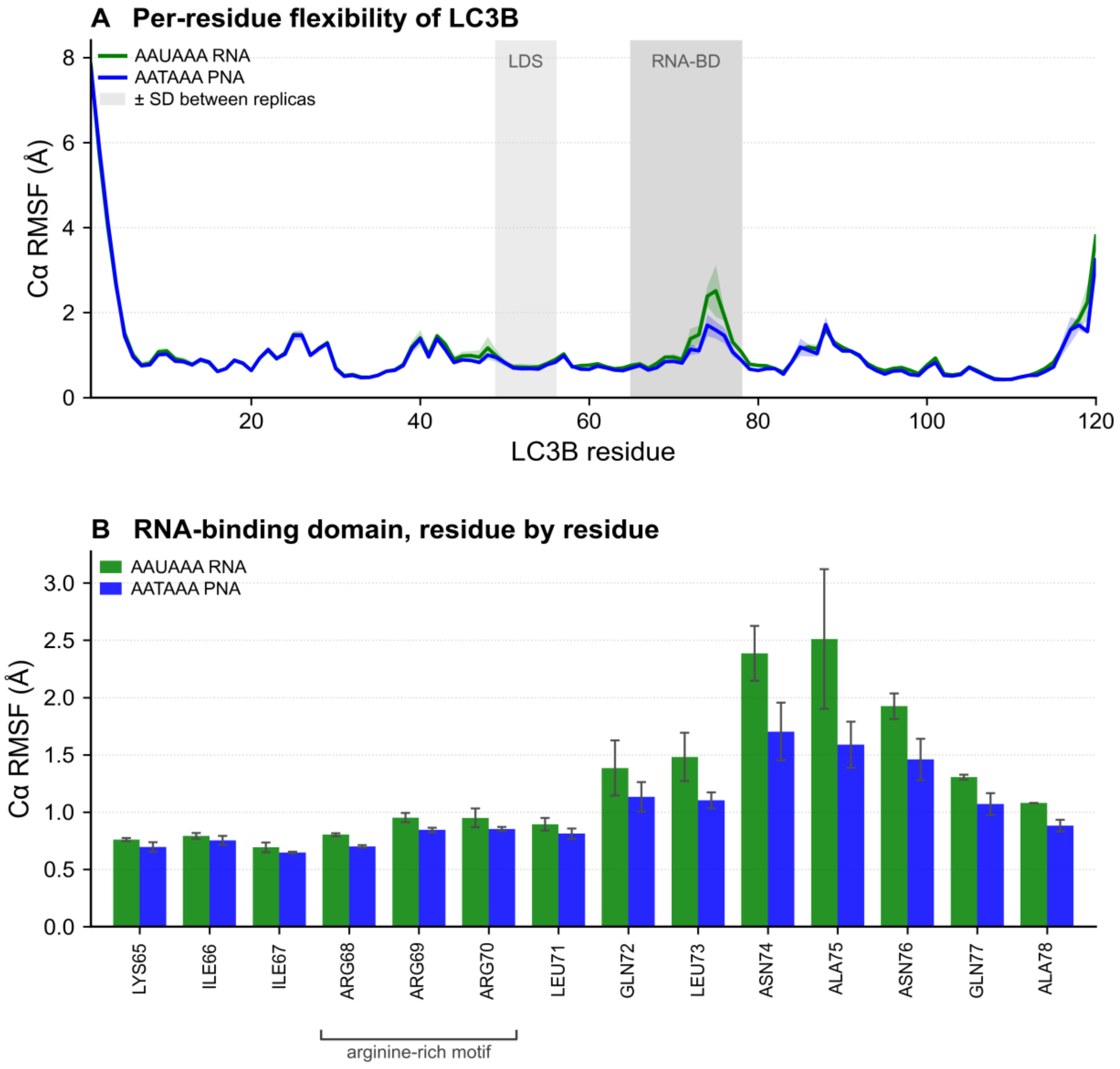

Figure S5. Per-residue LC3B Cα RMSF in complex with AAUAAA ssRNA and AATAAA PNA. (A) RMSF profile of the whole protein after superposition on the average structure; grey bands mark the LIR-docking site and the RNA-binding domain. (B) Residue-by-residue RMSF in the RNA-binding domain, with error bars representing the standard deviation between replicas and the bracket marking the R68/R69/R70 motif. The PNA systematically reduces fluctuations across the RNA-binding domain, especially in the N74–A78 flanking loop, while preserving the global protein flexibility profile.

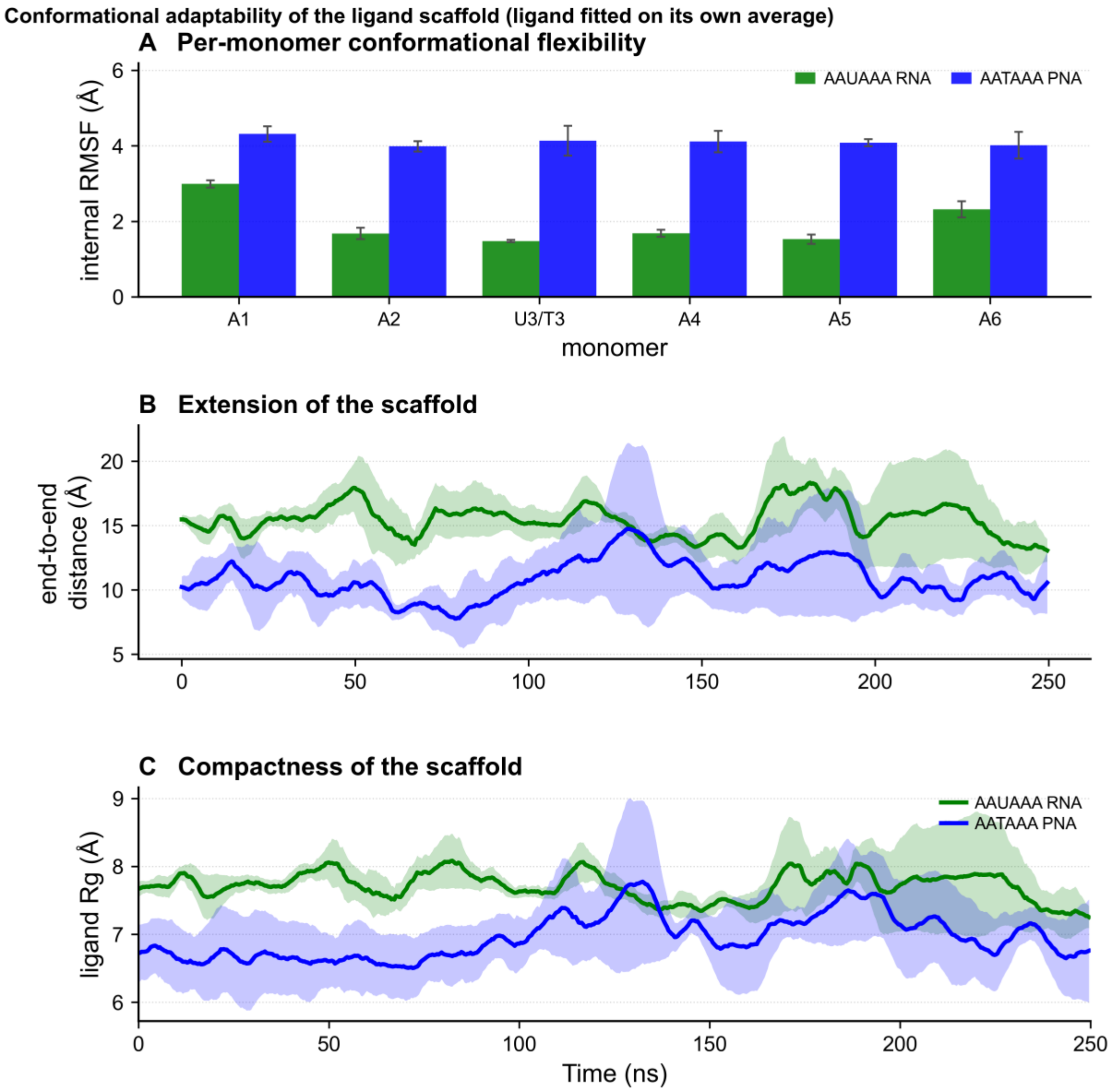

Figure S6. Flexibility and shape of the ligand scaffold in complex with LC3B. (A) Internal RMSF of each ligand monomer after superposition of the ligand onto its own average structure. (B) End-to-end distance, measured between the centroids of the first and last monomers, and (C) ligand radius of gyration as a function of simulation time. Lines report the mean over replicas, and shaded areas or error bars report the standard deviation between replicas. The AATAAA PNA is more internally flexible and adopts a more compact shape than the natural AAUAAA ssRNA, supporting the conformational adaptability proposed for simultaneous RNA-binding domain and LIR-docking site engagement.

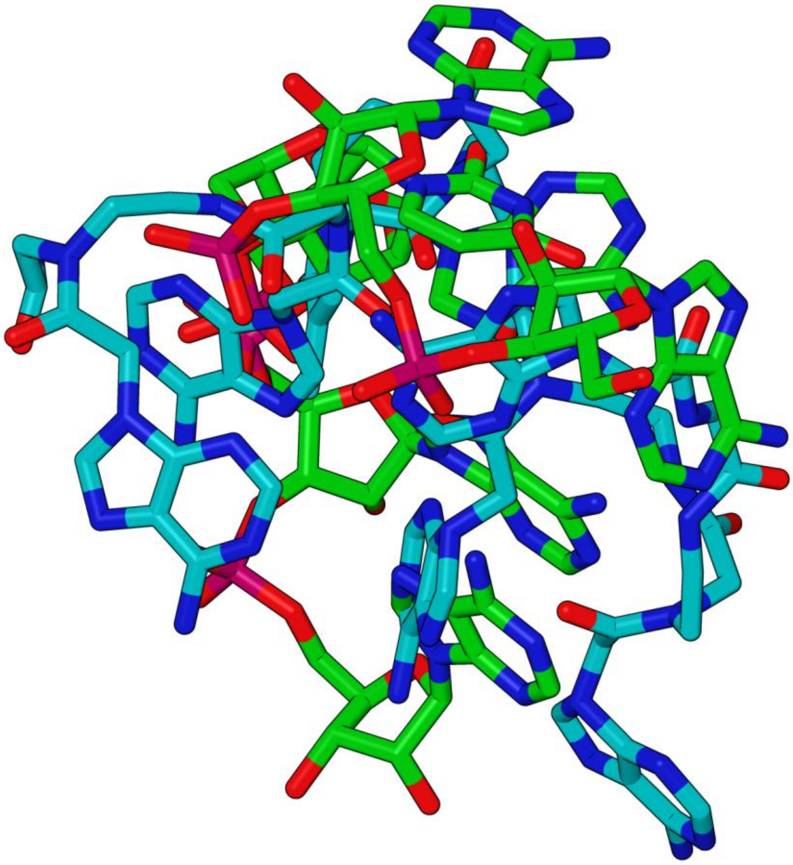

Figure S7. Structural overlap of the LC3B-bound AAUAAA RNA and AATAAA PNA poses. The AAUAAA ligand is shown in green and the AATAAA PNA in blue. The two ligands occupy a common region of the LC3B binding surface, indicating that the PNA preserves the overall recognition geometry of the natural AAUAAA sequence. The partial displacement of the AATAAA scaffold is consistent with its greater conformational adaptability and with the additional contacts formed around the LIR-docking site.

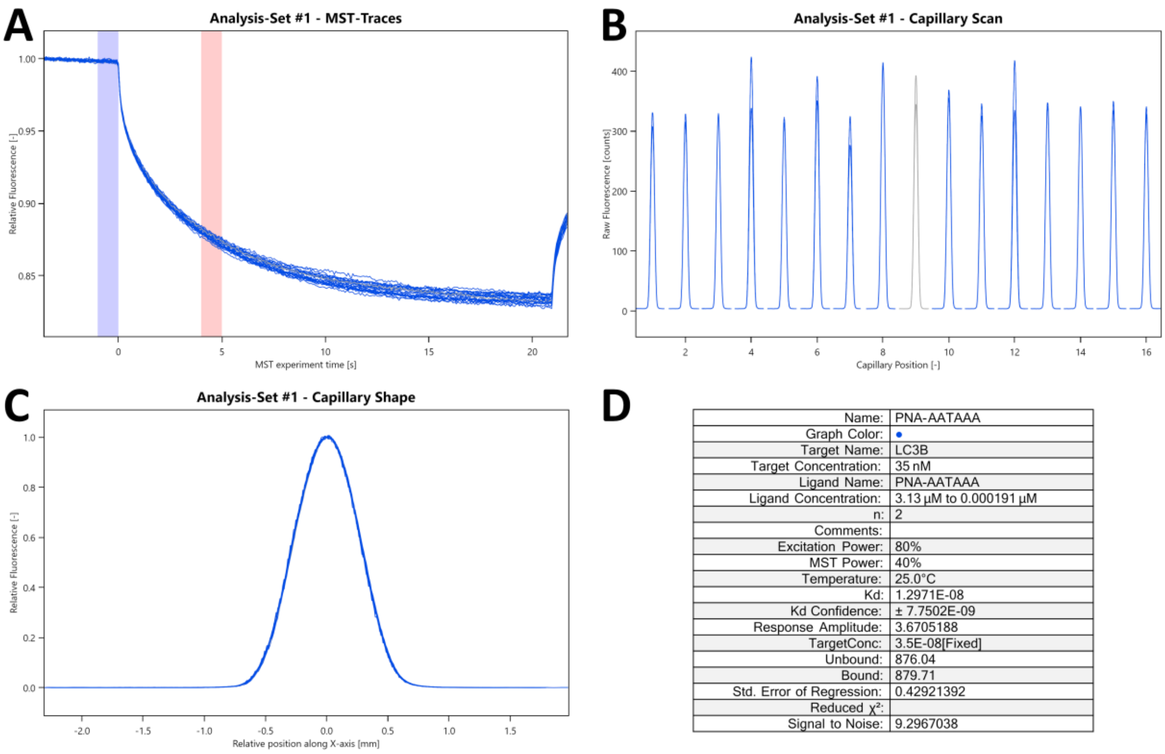

Name:	PNA-AATAAA
Graph Color:	•
Target Name:	LC3B
Target Concentration:	35 nM
Ligand Name:	PNA-AATAAA
Ligand Concentration:	3.13 µM to 0.000191 µM
n:	2
Comments:	
Excitation Power:	80%
MST Power:	40%
Temperature:	25.0°C
Kd:	1.2971E-08
Kd Confidence:	± 7.7502E-09
Response Amplitude:	3.6705188
TargetConc:	3.5E-08[Fixed]
Unbound:	876.04
Bound:	879.71
Std. Error of Regression:	0.42921392
Reduced χ^2:	
Signal to Noise:	9.2967038

Figure S8. MST binding affinity assay report of PNA-AATAAA on LC3B protein. (A) MST traces, (B) capillary scan, (C) capillary shape, and (D) dataset overview. The ninth concentration point (12.2 nM), identified as a clear outlier, is shown in grey.

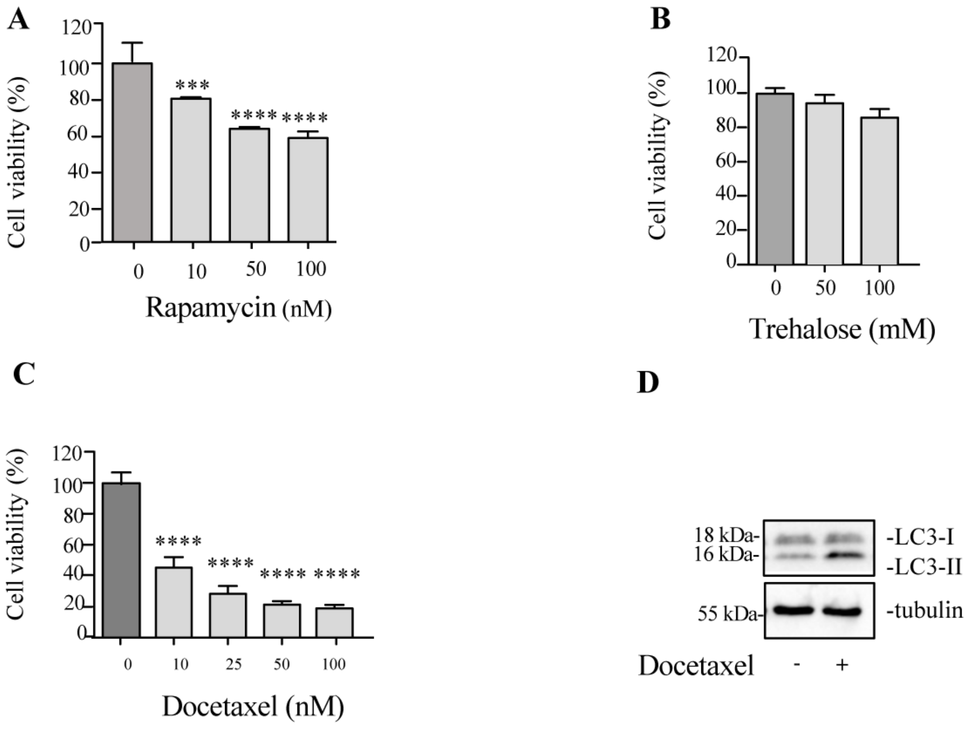

Figure S9. Effect of rapamycin, trehalose, and docetaxel on PC3 cell viability. (A-C) Cell viability was determined by MTT assay after 48 h of treatment with rapamycin, trehalose, and docetaxel on PC3 cells. Four independent biological samples were analyzed for each condition ($n = 4$). Statistical analysis was performed using one-way ANOVA followed by Dunnett's post hoc test (***$p < 0.001$ vs. C; ****$p < 0.0001$ vs. C). (D) Western blot of LC3 in PC3 cells treated with docetaxel (25 nM, 48 h). Tubulin expression was evaluated as a protein control.

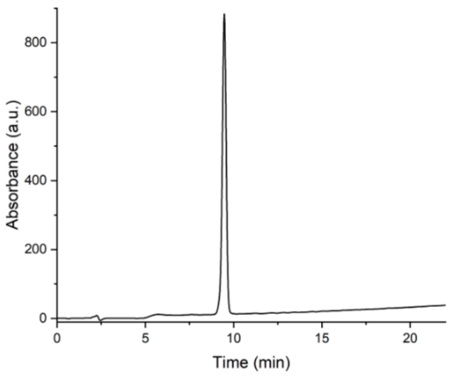

A

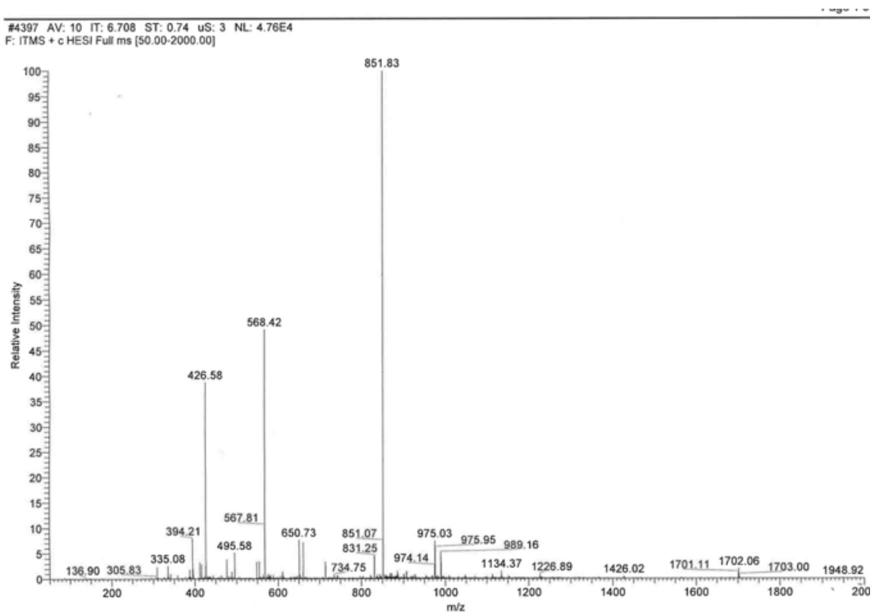

B

Figure S10. HPLC profile (A) and MS spectrum (B) of tested PNA AATAAA.
